# Programmable protein delivery with a bacterial contractile injection system

**DOI:** 10.1038/s41586-023-05870-7

**Published:** 2023-03-29

**Authors:** Joseph Kreitz, Mirco J. Friedrich, Akash Guru, Blake Lash, Makoto Saito, Rhiannon K. Macrae, Feng Zhang

**Affiliations:** 1 Howard Hughes Medical Institute, Cambridge, MA USA; 2 Broad Institute of MIT and Harvard, Cambridge, MA USA; 3 McGovern Institute for Brain Research at MIT, Cambridge, MA USA; 4 Department of Brain and Cognitive Science, Massachusetts Institute of Technology, Cambridge, MA USA; 5 Department of Biological Engineering, Massachusetts Institute of Technology, Cambridge, MA USA

**Keywords:** Bacteriophages, Applied microbiology

## Abstract

Endosymbiotic bacteria have evolved intricate delivery systems that enable these organisms to interface with host biology. One example, the extracellular contractile injection systems (eCISs), are syringe-like macromolecular complexes that inject protein payloads into eukaryotic cells by driving a spike through the cellular membrane. Recently, eCISs have been found to target mouse cells^1–3^, raising the possibility that these systems could be harnessed for therapeutic protein delivery. However, whether eCISs can function in human cells remains unknown, and the mechanism by which these systems recognize target cells is poorly understood. Here we show that target selection by the *Photorhabdus* virulence cassette (PVC)—an eCIS from the entomopathogenic bacterium *Photorhabdus asymbiotica*—is mediated by specific recognition of a target receptor by a distal binding element of the PVC tail fibre. Furthermore, using in silico structure-guided engineering of the tail fibre, we show that PVCs can be reprogrammed to target organisms not natively targeted by these systems—including human cells and mice—with efficiencies approaching 100%. Finally, we show that PVCs can load diverse protein payloads, including Cas9, base editors and toxins, and can functionally deliver them into human cells. Our results demonstrate that PVCs are programmable protein delivery devices with possible applications in gene therapy, cancer therapy and biocontrol.

## Abstract (web summary)

The tail fibre of an extracellular contractile injection system (eCIS) from *Photorhabdus asymbiotica* recognizes targets expressed on eukaryotic host cells, and can be reprogrammed to target specific organisms and cell types for delivery of novel protein payloads.

## Main

For endosymbiotic bacteria, it is often advantageous to secrete factors that modulate host biology in favour of symbiont fitness^4^. However, many such factors cannot readily pass through cellular membranes; this has led to the development of intricate systems that actively deliver payload proteins into cells^5^. One example is the contractile injection systems (CISs), a class of syringe-like nanomachines resembling bacteriophage tails^6,7^.

CISs are macromolecular complexes containing a rigid tube structure housed in a contractile sheath, which is anchored to a baseplate and sharpened by a spike protein^8–14^. Payloads are thought to load into the lumen of the inner tube behind the spike, form fusion proteins with the tube, or associate with the spike itself, which—upon target cell recognition—is forced through the membrane via sheath contraction^2,3,15–17^. This strategy has proved remarkably successful across the biosphere, as CISs have been shown to target organisms from all three domains of life^12,18,19^. CISs can be anchored to the bacterial membrane, resulting in a contact-dependent delivery system known as the type VI secretion system^8,20^ (T6SS), or can be attached to the thylakoid membrane in cyanobacteria (tCIS) to be activated during a cellular stress response^13^; finally, they can be produced as free complexes (eCISs) and released extracellularly to deliver payloads independent of the bacterial producer^21–24^. eCISs are distributed widely throughout bacteria and archaea, and have been shown to cluster into at least six subfamilies, of which only two contain characterized examples^21–23^. eCIS payloads have been shown to exhibit a variety of natural functions, including modulation of the host cytoskeleton^18,24^, DNA cleavage^1^, induction of metamorphosis^15,25^ and host toxicity^22,24,26^, indicating that these systems have been adapted for multiple biological niches. Recently, eCISs have been found to target mouse cells^1–3^, raising the possibility that these systems could be harnessed as protein delivery tools. However, eCIS activity has yet to be demonstrated in human cells, and the mechanism by which eCISs recognize target cells—a necessity if these systems are to be developed into targeted delivery devices—remains to be elucidated.

## Reconstitution and engineering of an eCIS

**Fig. 1:**
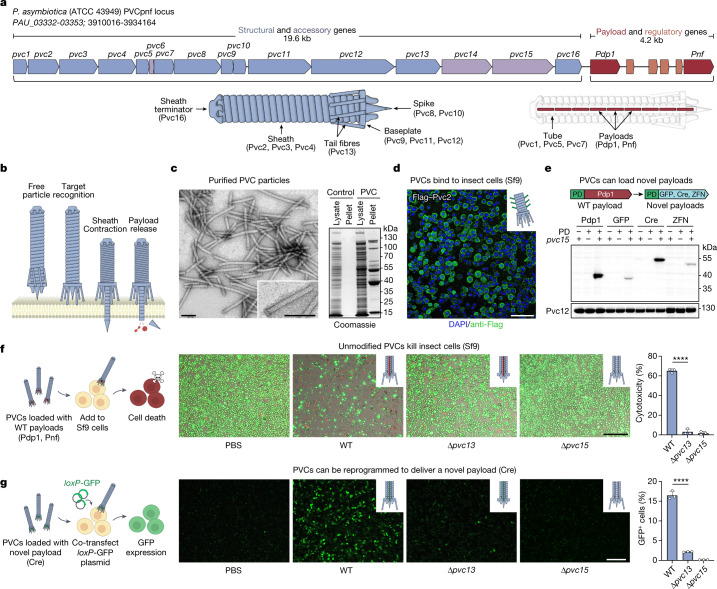
PVCs can be reprogrammed for custom protein delivery in eukaryotic cells. **a**, Schematic of the *P. asymbiotica* PVCpnf locus containing 16 structural and accessory genes (in blue and violet, respectively) followed by two payload genes (*Pdp1* and *Pnf*, in red) and four putative regulatory genes (in orange). PVC illustrations are approximations and are not drawn to scale. **b**, Proposed mechanism of PVC-mediated protein delivery. PVCs probably recognize target cells via tail fibres (Pvc13), leading to a contraction of the sheath mechanism that drives a spike through the cellular membrane. Payload proteins are then thought to enter the cell via disassembly of the spike–tube complex. **c**, PVCs can be purified from *E. coli*. The PVCpnf locus was transformed into *E. coli* and intact PVC particles were isolated. The resulting PVC preparations were then run on a denaturing SDS–PAGE gel and imaged with negative-stain TEM. Scale bars, 100 nm. **d**, PVCs can be visualized binding to target cells. PVC particles containing epitope-tagged sheath proteins (Pvc2) were incubated with Sf9 cells and binding was visualized via immunofluorescence. Scale bar, 100 μm. **e**, Non-native payloads can be loaded into PVC particles. The Pdp1 packaging domain (PD) (Extended Data Fig. 3b,c) was fused to novel proteins and loading was determined via denaturing western blot. Pvc12 (baseplate) served as a loading control. WT, wild-type. **f**, Wild-type PVC particles kill cultured insect cells. PVC-mediated cytotoxicity required both the action of the putative target recognition gene (*pvc13*; tail fibre) and payload loader (*pvc15*; ATPase). Scale bar, 200 μm. **g**, PVCs can deliver a non-native payload (loaded as in **e**) into target cells. Scale bar, 200 μm. Data in **f**,**g** are mean ± s.d. with *n* = 3 biological replicates; one-way ANOVA with Bonferroni post hoc test. ****P < 0.0001. Source data

For our studies of eCIS activity, we focused on one subtype of eCISs: the PVCs. PVCs are eCISs produced by members of the genus *Photorhabdus*, which exist as endosymbionts within entomopathogenic nematodes^24^. PVCs consist of an operon of approximately 20 kb containing 16 core genes (*pvc1–16*) that are necessary for the assembly of a functional injection system (Fig. 1a). Immediately downstream of *pvc1*–*16* are the payloads *Pdp1* and *Pnf*, which—as with all eCISs—are thought to enter target cells via contraction of the PVC sheath and subsequent disassembly of the spike–tube complex (Fig. 1b).

**Extended Data Fig. 1:**
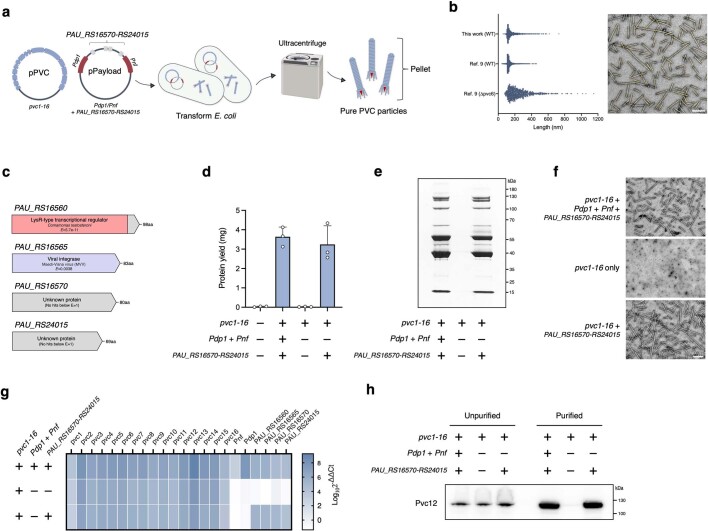
Analysis of PVC length and gene regulation. **a**, Workflow for PVC production in *E. coli*. The PVC locus was synthesized *de novo* and cloned into two separate plasmids: a structural/accessory plasmid (pPVC) containing *pvc1*-*16* and a payload/regulatory plasmid (pPayload) containing payloads to be delivered (*Pdp1*/*Pnf*) along with several genes thought to regulate PVC gene expression (*PAU_RS16570-RS24015*). **b**, Comparison of PVC length distributions from this manuscript and from a previous work^9^. A representative TEM image (with ROIs used to measure PVC lengths highlighted yellow) is also shown. Scale bar, 200 nm. **c**, HHpred/Pfam annotation of putative PVC regulatory genes (*PAU_RS16570-RS24015*). At least one gene (*PAU_RS16560*) contains a predicted domain known to be involved in gene regulation (LysR-type transcriptional regulator domain). **d**, Bulk protein yields after purification (from 500 mL of bacterial culture) with or without *PAU_RS16570-RS24015*. A roughly 100-fold increase in protein yield was observed when these genes were included alongside *pvc1-16*. Values are mean ± s.d. with *n* = 3 biologically independent reiterations of the purification procedure. **e**, SDS–PAGE gel depicting the results of purification with or without *PAU_RS16570-RS24015*, confirming the result from (d). **f**, TEM images depicting the results of purification with or without *PAU_RS16570-RS24015*, further confirming the result from (d), and indicating the resulting payload-deficient particles still retain a canonical structure. Scale bar, 200 nm. **g**, RT-qPCR analysis of PVC expression with or without *PAU_RS16570-RS24015*. No significant depletion of mRNA levels for *pvc1-16* were observed in cells lacking *PAU_RS16570-RS24015*, indicating these genes do not play a straightforward role as transcriptional regulators for this locus. Values are mean with *n* = 3 separate qPCR wells for each condition. **h**, Western blot against a structural PVC component (Pvc12) in the unpurified bacterial culture as well as a purified sample. A Pvc12 band was observed in bacteria lacking *PAU_RS16570-RS24015*, demonstrating these genes are not necessary for the expression of at least one PVC structural protein. This may suggest *PAU_RS16570-RS24015* affect the assembly (rather than expression) of PVC particles. Source data

**Extended Data Fig. 2:**
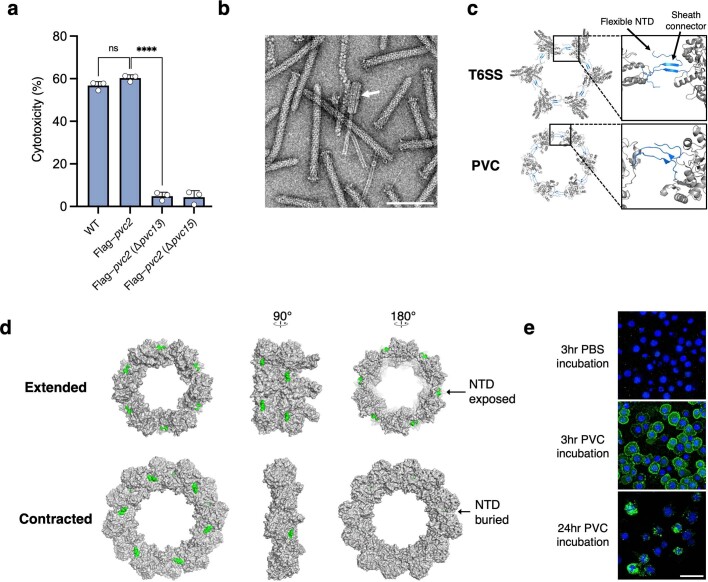
PVCs can be tagged with external epitope tags without loss of activity. **a**, PVCs harbouring Flag–Pvc2 are active in Sf9 cells, indicating sheath contraction (which is necessary for injection of target cells^7^) is likely not impaired by the addition of the Flag tag on Pvc2. Values are mean ± s.d. with *n* = 3 biological replicates. Statistical significance was computed using one-way ANOVA with Bonferroni post hoc test; ****P < 0.0001; ns, not significant. **b**, Contracted Flag-tagged PVCs can be observed in TEM, providing further evidence this tagging strategy does not inhibit sheath contraction. A PVC particle exhibiting a contracted phenotype is indicated with an arrow. Scale bar, 100 nm. **c**, The connector domain between sheath subunits for both T6SS (PDB: 3J9G) and PVC (PDB: 6J0B) contains a flexible N-terminal domain (NTD) that likely enables N-terminal Flag-tagging of this protein without loss of sheath contraction. **d**, The N-terminus of Pvc2 is externally exposed when the PVC is in the extended state (PBDs: 6J0B, 6J0C), enabling IF-based detection of PVC particles containing Flag–Pvc2. The first five residues of Pvc2 are colored green to mark their position in the sheath complex. **e**, IF signal against PVCs harbouring Flag–Pvc2 (green) decays after 24 h, possibly suggesting this IF method selectively stains for PVCs in the extended state. Scale bar, 50 μm. Source data

We first engineered *Escherichia coli* to produce PVCs from *P. asymbiotica* ATCC 43949 (PVCpnf) (Extended Data Fig. 1a) using a method similar to one described previously^9^. To facilitate downstream manipulation, we split the PVC system into separate structural and accessory (pPVC) and payload and regulatory (pPayload) plasmids. When examined using negative-stain transmission electron microscopy (TEM), the resulting protein complexes resembled canonical eCISs containing intact baseplates and sheath structures exhibiting a length of about 116 nm (Fig. 1c and Extended Data Fig. 1b). We observed that pPayload was necessary to produce detectable PVC particles (Extended Data Fig. 1c–h), suggesting that small genes in the payload region (labelled orange in Fig. 1a and hypothesized elsewhere^9^ to be involved in gene regulation) are critical for the formation of PVCs in *E. coli*. Finally, we also found that when these purified complexes were briefly exposed to cultured Sf9 insect cells (chosen owing to this cell line’s relation to the insect endogenously targeted by PVCpnf^24^), they bind robustly to the cell surface (Fig. 1d and Extended Data Fig. 2). These results demonstrate that *E. coli* can be used to manufacture PVC complexes exhibiting both proper assembly and targeting.

**Extended Data Fig. 3:**
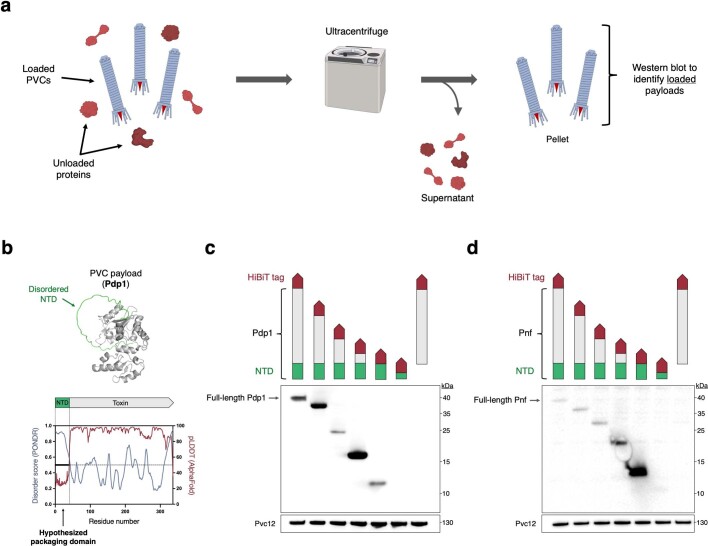
PVC payloads Pdp1 and Pnf are loaded via N-terminal packaging domains. **a**, Workflow for studying payload loading in PVCs. Only fully assembled PVC particles (along with any loaded payloads) localized to the pellet after ultracentrifugation; low molecular weight free proteins, however, did not pellet. This enabled the identification of loaded payloads via denaturing western blot of the pellet fraction. **b**, The PVC payload Pdp1 contains a predicted N-terminal packaging domain. The N-terminal domain (NTD) of Pdp1 is likely disordered as it exhibits a low pLDDT score in AlphaFold (it appears as a long disordered extension in the protein’s predicted structure) as well as a high PONDR VSL2 score^49^. This N-terminal disordered region could serve as a “packaging domain”—a molecular identifier to assist the PVC’s loading machinery in identifying and loading the proper payloads. **c-d**, N-terminal packaging domains of Pdp1 (c) and Pnf (d) are necessary and sufficient for loading of these proteins into a PVC particle. Pdp1 and Pnf were tagged with C-terminal HiBiT tags (to enable detection via western blot) and were serially truncated to locate minimal sequences capable of loading these payloads into a PVC particle. Domains on the N termini of each payload protein were sufficient for loading, and truncation of these NTDs inhibited loading, indicating the NTDs of Pdp1 and Pnf serve as packaging domains for this PVC. As in Fig. 1e, the baseplate protein (Pvc12) was chosen as a loading control for this western blot as an equivalent number of Pvc12 units are found in each PVC particle. Note that in panel (d), while both the Pvc12 and Pnf bands were visualized on the same gel, two different exposure times were used (to avoid oversaturation of the brighter Pvc12 bands).

To develop PVCs into programmable protein delivery devices, we next attempted to load novel, non-native payloads into the PVC (Fig. 1e and Extended Data Fig. 3a). Although the mechanism by which PVCs recruit payloads is not fully understood, it has been recently shown that highly disordered regions on the N termini of endogenous PVC payload proteins (Extended Data Fig. 3b) are involved in the loading process^3^. We confirmed that modified payloads lacking this disordered region did not load into PVCs (Extended Data Fig. 3c,d), indicating that this region represents a ‘packaging domain’ that is necessary for loading a payload into the PVC complex. We thus fused this packaging domain to various proteins that are not naturally loaded into the PVC (GFP, Cre and a zinc finger nuclease) and tested whether the resulting engineered payloads were loaded into the PVCs (Fig. 1e). We found that in the presence of *pvc15* (an ATPase also shown to be necessary for payload loading^3^), all three novel payloads co-purified with the PVCs, confirming that this method (N-terminal fusion of a packaging domain) is a generalizable strategy for loading novel proteins into PVC particles.

**Extended Data Fig. 4:**
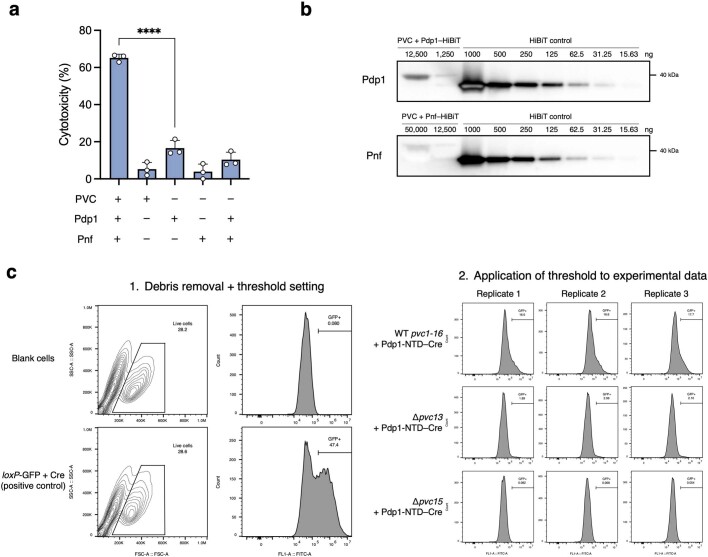
Both PVCs and payloads are necessary for efficient killing of insect cells. **a**, Unloaded PVCs, as well as separately purified payloads (toxins Pdp1 and Pnf), are insufficient to produce efficient cytotoxicity in Sf9 cells. Pdp1 and Pnf were purified independently of PVC complexes by affinity purification and were administered to Sf9 cells at doses roughly equivalent to the amount loaded into PVCs (determination of biologically-relevant payload doses in (b)). Efficient cytotoxicity was observed only when both the PVC complex and the payload proteins were co-purified. Values are mean ± s.d. with *n* = 3 biological replicates. Statistical significance was computed using one-way ANOVA with Bonferroni post hoc test; ****P < 0.0001. **b**, Dose determination for cytotoxicity assay from (a). PVCs loaded with HiBiT-tagged payload proteins were run alongside a titration of a control protein (HiBiT control protein; Promega N3010); band intensities (quantified with ImageJ) were then compared to determine the approximate quantity of payload protein loaded into PVCs. From this analysis, we observed that Pdp1 is loaded in higher quantities than Pnf (~1% and ~0.1% of total protein, respectively); we thus dosed Sf9 cells in (a) with purified Pdp1 and Pnf protein at 1% and 0.1% of the dose of loaded PVCs. **c**, Representative flow cytometry analysis of PVC-mediated protein delivery in cultured cells. After gating the cells on FFC/SSC to remove debris, we set a GFP-/+ threshold using a positive control condition (cells transfected with both *loxP*-GFP and Cre). We then applied this threshold to experimental data to determine the proportion of cells expressing GFP. Source data

Finally, we tested whether PVC-mediated protein delivery—with both endogenous and engineered payloads—could be directly observed in cultured insect cells. After incubating Sf9 cells with unmodified PVCs harbouring native toxin payloads, we observed robust cytotoxicity (Fig. 1f). Notably, we found that this phenotype required the presence of several critical PVC genes, including what we hypothesized to be the targeting element of the PVC (*pvc13*, encoding the tail fibre) and a gene previously suggested to be the payload loader^3^ (*pvc15*). Additionally, administration of separately purified payloads or unloaded PVC complexes was insufficient to reproduce this phenotype (Extended Data Fig. 4a,b), indicating that the observed activity required the actions of both the PVC complex and the toxin payloads. Furthermore, when administered to Sf9 cells harbouring a Cre reporter system (*loxP*–GFP), PVCs artificially loaded with Cre (using the method described in Fig. 1e) produced GFP signal (Fig. 1g and Extended Data Fig. 4c), demonstrating that a novel protein payload can be functionally delivered via the PVC. Together, these results show that recombinant PVCs are biologically active against cultured insect cells and can be reprogrammed to both load and deliver non-native proteins into target cells to yield novel biological activities.

## Altering PVC tropism towards human cells

**Extended Data Fig. 5:**
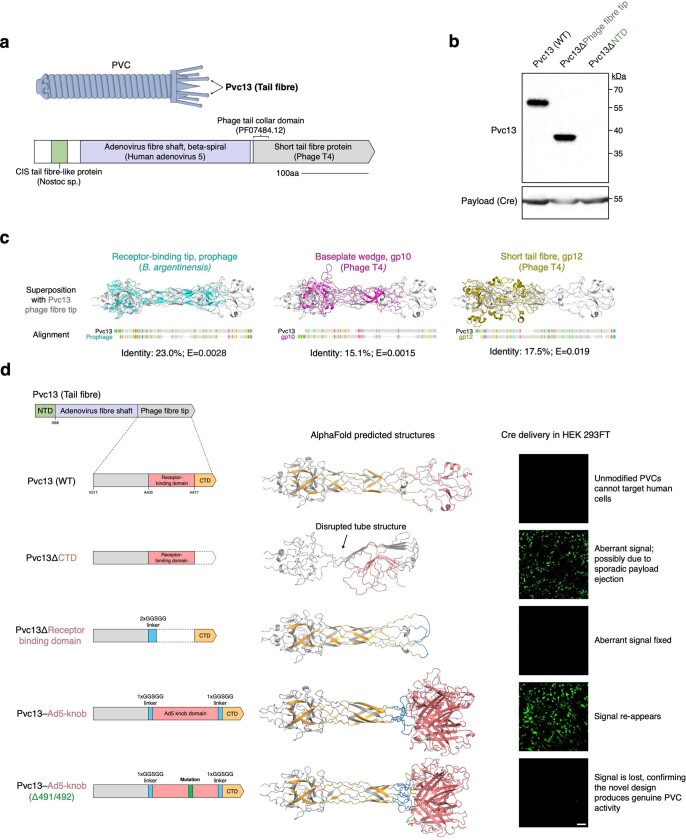
AlphaFold-guided design of modified tail fibres for use in retargeting PVCs. **a**, Domain organization of the PVC tail fibre gene (*pvc13*). The tail fibre contains an N-terminal domain (NTD) with homology to tail fibres from other CISs, a domain that maps to shaft domains of adenoviral fibres, and a C-terminal domain with homology to the host-binding domains of bacteriophage tail fibres. Domains were depicted based on statistically-significant HHpred hits (>95%) and are drawn roughly to scale. Scale bar, 100 aa. **b**, Pvc13 is loaded via the NTD, implicating the C-terminal phage fibre tip domain as the cell-binding domain. Pvc13 was truncated at either end and loading was determined via denaturing western blot on purified PVC particles. Only truncation of the NTD resulted in a loss of Pvc13, suggesting this domain connects the tail fibre to the PVC. An additional blot against the payload (Pdp1-NTD–Cre) was included to confirm the presence of assembled PVCs. **c**, The C-terminal phage fibre tip domain of Pvc13 shares structural and sequence similarity with known receptor-binding domains from bacteriophages. Structural superpositions were generated between the phage fibre tip domain from Pvc13 (in grey) and analogous regions from a prophage tail fibre in *Bizionia argentinensis* (cyan; PDB: 6OV6), gp10 from phage T4 (magenta; PDB: 5IV5) and gp12 from phage T4 (yellow; PDB: 5HX2). **d**, Predicted structures and delivery characteristics of wild-type and engineered PVC tail fibres. The AlphaFold predicted structure of the C-terminal phage fibre tip domain contains a helical tube structure with a globular tip on one end that we hypothesized to be the distal target recognition domain of the overall tail fibre. Importantly, there also exists a short 32 aa region (labelled CTD; depicted in gold) that loops back through the tube, likely stabilizing it. We observed that designs lacking this CTD (when co-purified with the PVC complex) produced misleading activity in Cre delivery experiments in HEK 293FT, perhaps as a result of sporadic ejection of the Cre payload from the PVC particle (free Cre protein can enter cells independently of a delivery vehicle^50^). However, designs retaining this CTD produced no aberrant signal in HEK 293FT, indicating payload ejection was once again properly regulated. Amino acid sequences for representative Pvc13 designs can be found in Supplementary Tables 1–4. Scale bar, 150 μm.

The mechanism by which PVCs bind to target cells is not known. However, target recognition by contractile tail phages (which resemble PVCs) is well understood. Phage T4 possesses six long tail fibres that extend from the baseplate complex and form reversible interactions with lipopolysaccharide molecules or outer membrane proteins on the surface of host cells^27–29^. This process positions the phage in the correct orientation above the target cell and enables the baseplate to move close enough to the surface of the cell to bind irreversibly and initiate injection of the phage genome into the cell^29,30^. A number of studies have demonstrated that modifications to the tail fibres of phages and other bacteria-targeting CISs are sufficient to alter target specificity in predictable ways^31–34^, indicating that these proteins are important determinants of target specificity in these systems. Although it is not currently known how PVCs and other eCISs target cells and initiate the injection process, we proposed that it may be possible to alter PVC target specificity using a similar technique. In particular, PVC loci contain a tail fibre gene (*pvc13*) that possesses a predicted domain similar to the receptor-binding tip from the short tail fibre of phage T4 (Extended Data Fig. 5a). Of note, PVC tail fibres diverge from phage tail fibres in that they often also contain regions that map to receptor-binding proteins from eukaryotic viruses (especially those of adenoviruses), supporting the hypothesis that the PVC tail fibre is involved in the recognition of a eukaryotic organism. PVC tail fibres have also been shown to connect to the baseplate and fold upwards along the sheath in a similar fashion as in phages^9^. Overall, these observations suggest that the tail fibre is probably involved in target recognition and could be harnessed to manipulate the target specificity of PVCs.

**Fig. 2:**
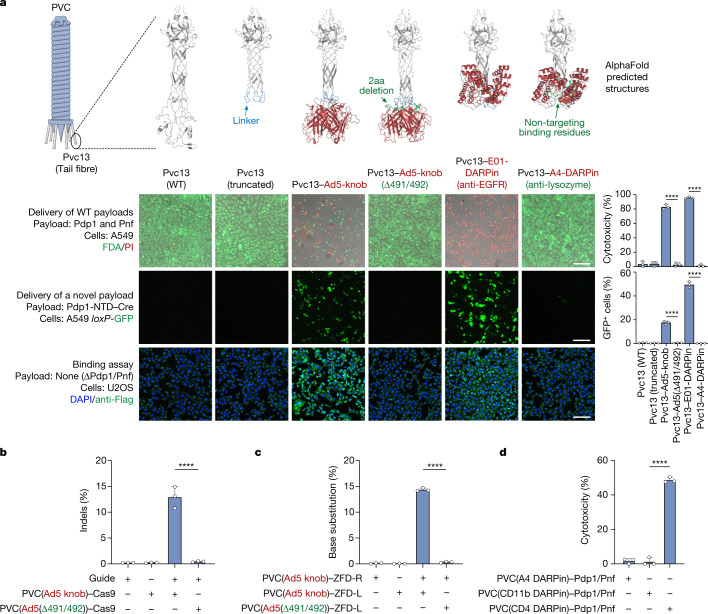
Reprogramming PVCs for injection of human cells. **a**, AlphaFold-guided engineering of Pvc13 (tail fibre) results in PVCs exhibiting efficient delivery activity in human cells. Top, the putative target recognition domain of Pvc13 (amino acids 403–476) was deleted (producing Pvc13 (truncated)) and replaced with either the target-binding domain from human adenovirus 5 (producing Pvc13–Ad5-knob) or a DARPin specific for human EGFR (producing Pvc13–E01-DARPin). Pvc13 variants containing non-targeting binding domains (Pvc13–Ad5-knob(Δ491/492) and Pvc13–A4-DARPin) were also included as negative controls. Bottom, PVC particles were loaded with wild-type (WT) toxin payloads or with a novel payload (Cre), and PVC-driven protein delivery was measured as cytotoxicity or GFP expression, respectively. Bottom row, novel PVC designs can be observed binding to human cells (in this case U2OS to facilitate immunofluorescence imaging). Binding of target cells required the presence of Pvc13–Ad5-knob or Pvc13–E01-DARPin; PVCs harbouring wild-type Pvc13, truncated Pvc13 or a non-targeting fusion protein did not cluster on the cell surface. Scale bars, 300 μm. **b**, Human-targeting PVC designs can deliver SpCas9 protein into HEK 293FT cells, enabling PVC-mediated gene editing in human cells. Indel formation required the presence of an unmutated Ad5 knob domain in Pvc13, indicating that this activity required the action of the PVC. Conditions are listed in the format PVC(Pvc13 design)–payload. **c**, Human-targeting PVC designs can deliver ZFDs into HEK 293FT cells, enabling base editing in human cells. On-target G-to-A base substitution was observed only when each ZFD half (ZFD-L and ZFD-R) was delivered by a properly targeted PVC; non-targeting PVCs produced negligible activity. **d**, Human-targeting PVC designs can kill leukaemia cells (Jurkat). Cytotoxicity was produced only when PVCs were retargeted towards a T cell receptor known to be expressed by Jurkat cells (CD4), but not when retargeted towards a myeloid receptor not found on Jurkat cells (CD11b). Data are mean ± s.d. with *n* = 3 biological replicates; one-way ANOVA with Bonferroni post hoc test. Source data

**Extended Data Fig. 6:**
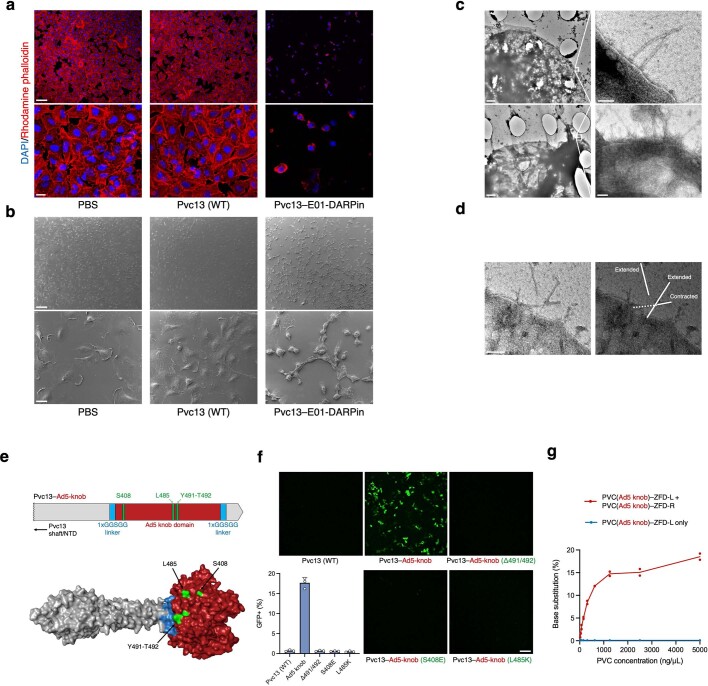
High-resolution imaging of PVC-mediated binding and killing of human cells. **a**, A549 cells exhibit F-actin condensation after exposure to EGFR-specific PVCs (harbouring Pvc13–E01-DARPin), indicating the endogenous PVC toxin payloads (which are known to target the actin cytoskeleton^24^) were successfully delivered. Scale bars, 100 μm (upper row) and 20 μm (lower row). **b**, SEM images of A549 cells treated with the same PVC designs as in (a). Scale bars, 100 μm (upper row) and 20 μm (lower row). **c**, EGFR-specific PVCs can be directly visualized binding to A549 cells. Scale bars, 1 μm (left panels) and 100 nm (right panels). **d**, Contracted PVCs are visible on the cell surface, suggesting PVC-mediated protein delivery in human cells is mediated by sheath contraction. Scale bar, 100 nm. **e**, Locations of additional nonbinding mutations within the AlphaFold predicted structure of Pvc13–Ad5-knob. We screened two additional mutants—S408E and L485K—each containing single amino acid substitutions shown previously to inhibit the binding of Ad5 to target cells^51^. **f**, New Ad5 knob mutants are unable to retarget PVCs toward HEK 293FT cells. PVCs equipped with Pvc13–Ad5-knob (S408E or L485K) were loaded with Cre and administered to HEK 293FT cells harbouring a *loxP*-GFP plasmid (as in Fig. 2a); only PVCs equipped with tail fibres containing wild-type Ad5 knob domains produced efficient GFP expression. Values are mean ± s.d. with *n* = 3 biological replicates. Scale bar, 150 μm. **g**, Titration of PVCs harbouring Pvc13–Ad5-knob over HEK 293FT cells. Cells were exposed to a dilution series of Pvc13–Ad5-knob particles loaded with either ZFD-L or ZFD-R (the same particles as those in Fig. 2c) and PVC-mediated base substitution was measured with deep sequencing. PVC efficiency saturated at about 1,000 ng/µL. Values are mean with *n* = 2 biological replicates. Source data

We tested whether modifications to the PVC tail fibre protein (Pvc13) could produce alterations to tropism and enable targeting of human cells. We used AlphaFold^35–37^ to predict the 3D structure of the putative distal tip of Pvc13—the region that we predicted would make the initial contact with target cells (Fig. 2a and Extended Data Fig. 5b–d). When queried as a trimer, the C-terminus of Pvc13 forms a predicted helical tube structure with a globular tip that we believe to be the binding domain of the overall tail fibre. We hypothesized that altering the binding characteristics of this distal binding domain could result in predictable changes to PVC tropism, as is the case with tail fibres from other CISs. To test this, we inserted a novel binding domain specific for human cells (the trimeric knob domain from human adenovirus 5 (Ad5)^38^ or the epidermal growth factor receptor (EGFR)-specific designed ankyrin repeat protein (DARPin) E01^39^) into the putative C-terminal binding region of Pvc13 (to generate Pvc13–Ad5-knob or Pvc13–E01-DARPin, respectively) and tested whether the resulting PVCs could target human cells. For this experiment, we used A549 human lung adenocarcinoma cells as a model cell line as it is known to overexpress EGFR and is sensitive to Ad5 infection. We found that PVCs equipped with Pvc13–Ad5-knob or Pvc13–E01-DARPin efficiently killed A549 cells when loaded with native toxins Pdp1 and Pnf (Fig. 2a and Extended Data Fig. 6a–d) or produced efficient Cre-driven GFP expression in A549 *loxP*-GFP cells when loaded with Pdp1-NTD–Cre (the N-terminal domain (NTD) of Pdp1 tethered to Cre) as described in Fig. 1e (Fig. 2a). Notably, this activity was abolished when PVCs were equipped either with mutant Ad5 knob domains (Δ491/492—previously shown to reduce binding of Ad5 to target cells^40^; Extended Data Fig. 6e,f) or a non-targeting DARPin (anti-lysozyme DARPin A4 (Protein Data Bank: 5OP1)), indicating that PVC activity in human cells is dependent on the presence of tail fibres that can properly bind human cells. Finally, we found that PVCs harbouring Pvc13–Ad5-knob or Pvc13–E01-DARPin clustered on the surface of human cells (Fig. 2a, bottom), suggesting that the observed activity was the result of a novel binding interaction between the engineered PVCs and target cells. These results demonstrate that Pvc13 is a tropism-determining element of the PVC and that this protein can be modified to yield predictable changes to the target specificity of this system.

To further characterize the PVC as a protein delivery tool, we extended the results of Fig. 2a by establishing several useful delivery applications in human cells. We first tested whether PVCs could be reprogrammed to load and deliver *Streptococcus pyogenes* Cas9 (SpCas9) to effect gene editing in human cells (Fig. 2b). We found that when PVCs retargeted with Pvc13–Ad5-knob were loaded with Cas9 (using a similar strategy as in Fig. 1e), the resulting particles produced on-target insertions and deletions (indels) in HEK 293FT cells harbouring a guide RNA. This experiment is notable because Cas9 is much larger than the payloads natively loaded by this PVC (170 kDa for Pdp1-NTD–Cas9 versus 37 kDa for Pdp1 and Pnf), demonstrating that PVCs can deliver diverse payloads of varying sizes (supporting similar conclusions from other studies^3,22^). To achieve guide RNA-free gene editing with PVCs, we next attempted to deliver zinc finger deaminases (ZFDs), a recently described system consisting of zinc finger domains tethered to split deaminases^41^. When PVCs retargeted with Pvc13–Ad5-knob were loaded with either the left or right arm of a ZFD targeting the human TRAC locus (ZFD-L or ZFD-R, respectively) and were co-administered to HEK 293FT cells, we observed on-target G-to-A base substitution (Fig. 2c and Extended Data Fig. 6g), indicating that PVCs can deliver ZFDs to effect base editing in human cells. Finally, inspired by the endogenous biological function of PVCs (targeted killing via delivery of toxins), we tested whether PVCs could be used to specifically kill human cancer cells. We found that PVCs loaded with endogenous toxins (Pdp1 and Pnf) produced efficient cytotoxicity in Jurkat cells when they were retargeted with a DARPin specific for a T cell receptor (CD4; Fig. 2d). Notably however, PVCs targeting a myeloid receptor not produced by Jurkat cells (CD11b) resulted in negligible cell death, suggesting that PVC-mediated cytotoxicity in human cancer cells is receptor-specific.

## Interrogation of PVC target specificity

One notable characteristic of bacteriophages is their narrow target specificity^42^. Phage specificity is thought to be conferred by highly evolved binding interactions between phage tail fibres and receptors displayed by host bacteria^27,43^. Although this can make the treatment of bacterial infections with phages challenging, specificity is a critical feature of modern targeted therapeutics and is essential for the treatment of cancer and genetic disease. Our discovery that PVC specificity is conferred by the tail fibre and that PVC tropism can be shaped via rational modification of this protein raises the possibility that PVCs (similar to phages) also exhibit a high degree of target specificity.

**Fig. 3:**
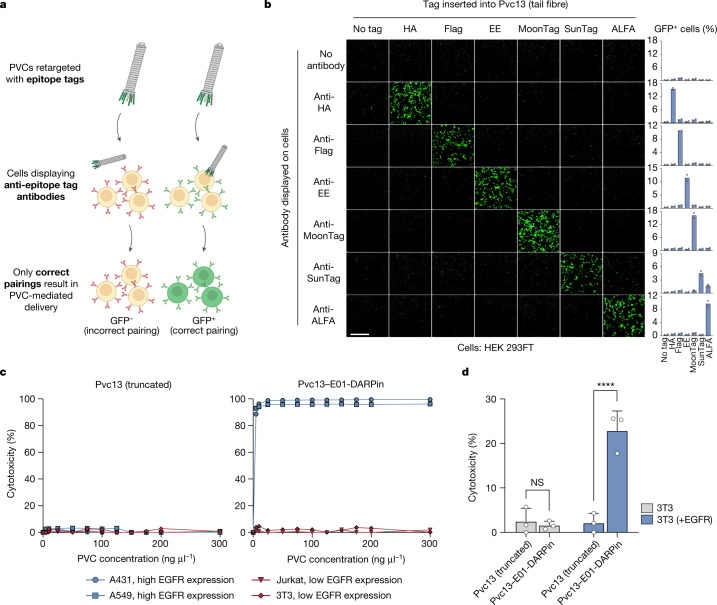
PVC-mediated protein delivery is highly specific. **a**,**b**, Specificity assay for PVCs based on artificial receptors. **a**, Schematic of the experiment. A panel of epitope tags was inserted into the distal binding domain of the tail fibre (amino acids 403–476 of Pvc13), and a panel of the associated receptors (anti-epitope tag antibodies) was displayed on the surface of HEK 293FT cells. As in Figs. 1g and 2a, PVC activity was measured as GFP expression following delivery of Cre into cells harbouring *loxP*-GFP. **b**, Only correct epitope–antibody pairings resulted in efficient PVC-mediated delivery of Cre, indicating that PVC activity requires a specific interaction between the tail fibre and the target cell. Scale bar, 500 μm. **c**, Specificity assay for PVCs based on an endogenous receptor. Right, PVCs loaded with Pdp1 and Pnf were retargeted towards human EGFR (with Pvc13–E01-DARPin, as in Fig. 2a) and administered to EGFR^+^ (A431 and A549) and EGFR^−^ (Jurkat and 3T3) cell lines. Cytotoxicity was observed only in the EGFR^+^ cell lines, and only when Pvc13 contained the anti-EGFR DARPin. No cytotoxicity was observed with PVCs harbouring truncated Pvc13 lacking a binding domain (amino acids 403–476) (left). **d**, Display of a target receptor is sufficient to sensitize cells to PVCs. A cell line (3T3) immune to PVC delivery (**c**) was transfected with a plasmid containing human EGFR and was exposed to EGFR-targeting PVCs; this time, the cells exhibited a loss of viability. Data are mean (**b,c**) or mean ± s.d. (**d**) with n = 2 (**b,c**) or n = 3 (**d**) biological replicates; two-way ANOVA with Bonferroni post hoc test. NS, not significant. Source data

To study PVC target specificity, we first constructed a panel of artificial HEK 293FT-derived cell types displaying defined non-native receptors that could be easily targeted by engineered PVCs (Figs. 3a,b). For simplicity, we chose as receptors a panel of antibodies (scFvs and nanobodies) specific for commercial epitope tags. We then inserted the associated panel of epitope tags into the distal binding domain of the tail fibre (as in Fig. 2a) and administered the resulting modified PVCs to these ‘cell types’ to understand how effectively PVCs undergo target selection. We found that PVCs retargeted with epitope tags were only capable of efficiently delivering payloads into cells displaying the appropriate binding partners for those epitope tags (Fig. 3b). This result indicates that PVC specificity is largely conferred by the interaction between the tail fibre and its target receptor, and that this interaction can be engineered to enable specific recognition of novel cell types.

We next assessed PVC specificity against EGFR, a natural receptor found endogenously on some human cell types (Fig. 3c). In this experiment, we tested whether a PVC programmed to target EGFR specifically targets cells known to express EGFR. We found that PVCs retargeted with an anti-EGFR DARPin (E01) and loaded with toxins (Pdp1 and Pnf) were only capable of efficiently killing EGFR^+^ cell lines (A549 and A431) and not EGFR^−^ cell lines (Jurkat and 3T3). In addition, we found that transfection of EGFR into an EGFR^−^ cell line from the previous experiment (3T3) sensitizes these cells to this PVC (Fig. 3d), indicating that the presence of an appropriate target receptor is sufficient to enable PVC-mediated delivery. These results—in addition to the specificity assay with artificial receptors in Fig. 3a,b—provide evidence that PVCs exhibit a high degree of target specificity and can only efficiently deliver payloads into cells displaying a suitable target receptor.

## In vivo protein delivery with PVCs

**Fig. 4:**
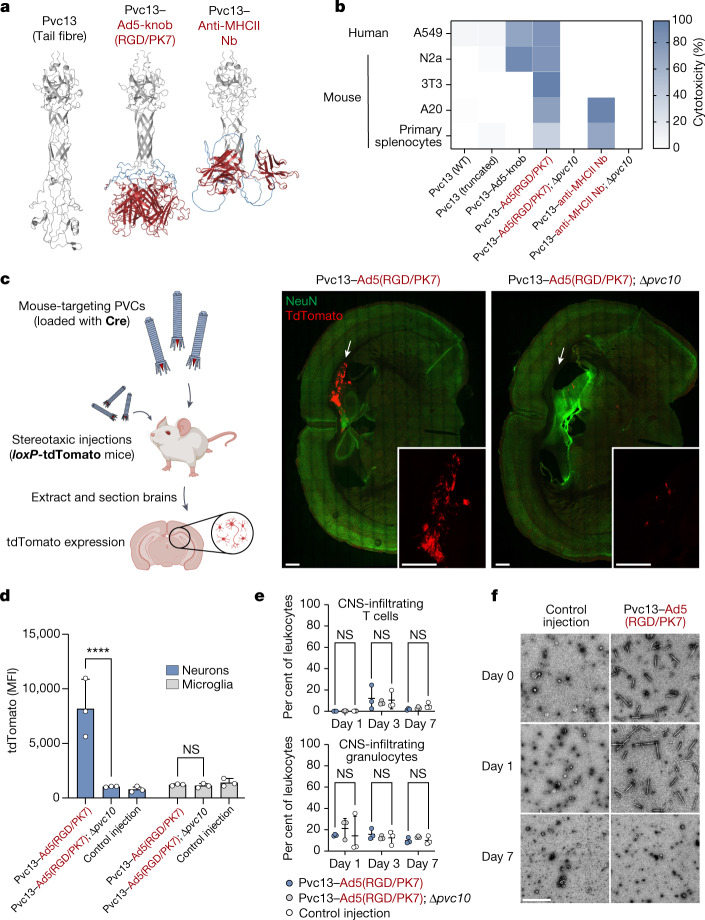
Reprogramming PVCs to achieve targeted delivery in mice. **a**, AlphaFold-predicted structures of novel mouse-targeting Pvc13 (tail fibre) designs. We replaced the wild-type binding domain of Pvc13 with an expanded tropism variant of the Ad5 binding domain (Ad5-knob(RGD/PK7)) or a nanobody (Nb) targeting mouse MHC class II protein (MHCII). **b**, Novel mouse-targeting PVC designs exhibit enhanced activity across mouse cell lines and primary cells. PVCs deficient for the spike tip protein (Pvc10) were used as negative controls for both novel Pvc13 designs. **c**, Left, schematic showing protein delivery in the mouse brain with PVCs. Right, PVCs equipped with Pvc13–Ad5-knob(RGD/PK7) produce tdTomato signal in the hippocampus of Ai9 (*loxP*-tdTomato) mice. Fluorescence was abolished when the spike tip protein (Pvc10) was deleted, confirming that the observed activity was mediated by the PVC. Injection sites are shown with white arrows. Scale bars, 500 μm. **d**, PVCs target neurons in vivo. Intracranial injections were performed as in **c** and single-cell extracts from treated brains were analysed with flow cytometry. The flow cytometry gating scheme is shown in Extended Data Fig. 8a. MFI, mean fluorescence intensity. **e**, Intracranial PVC injections do not induce immune cell migration to the CNS. The flow cytometry gating scheme is shown in Extended Data Fig. 8d. **f**, PVCs are transient in the mouse brain. Intact particles can be readily purified from treated mouse brains after 0 or 1 day, but not after 7 days, indicating that PVCs do not persist in brain tissues for extended periods. Scale bar, 500 nm. Data are mean (**b**) or mean ± s.d. (**d**,**e**) with *n* = 2 (**b**) or *n* = 3 (**d**,**e**) biological replicates; two-way ANOVA with Bonferroni post hoc test (**d**,**e**). Source data

To understand whether PVCs could eventually be used in humans, we next attempted to deliver proteins in a live mouse. To produce PVC variants that target mouse cells, we again used AlphaFold-guided engineering of Pvc13 (Fig. 4a). We screened two new binding domains: (1) a modified Ad5 knob domain (Ad5-knob(RGD/PK7)) that was previously used^44^ to expand the host range of Ad5 to mouse tissues, and (2) a nanobody targeting a mouse receptor^45^ (MHC class II). After equipping Pvc13 with these new binding domains, the resulting PVCs produced greatly enhanced activity in mouse cell lines and primary cells (Fig. 4b). Notably, we observed that although PVCs retargeted with Ad5-knob(RGD/PK7) exhibited broad tropism (as is true of Ad5 RGD/PK7 viruses^44^), PVCs targeting MHC class II showed a strong preference for MHC^+^ immune cells, providing further evidence that PVC activity is dependent on the presence of a suitable target receptor.

**Extended Data Fig. 7:**
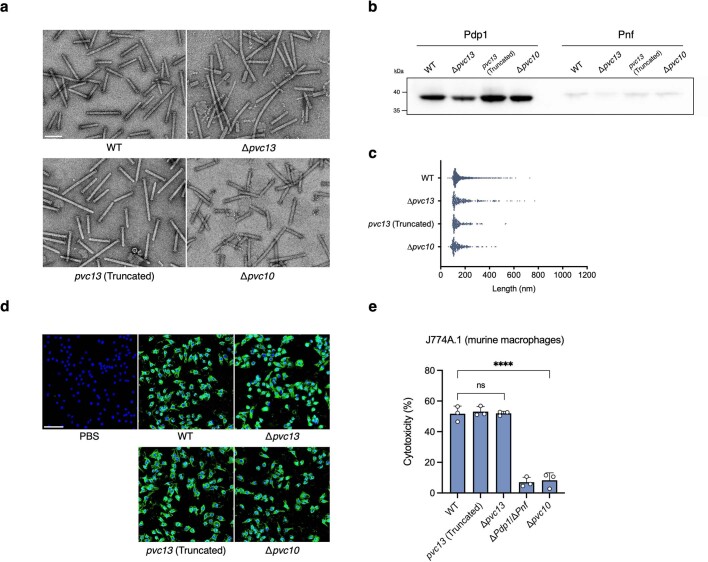
Properties of PVCs containing mutant tail fibre (Pvc13) or spike tip (Pvc10). **a**, PVCs harbouring deleted *pvc13* (as was used in Fig. 1f, g), truncated *pvc13* (lacking aa 403-476, the binding domain characterized in Fig. 2a), or deleted *pvc10* (as was used in Fig. 4b–e) all still form intact particles under TEM, indicating these genes are not necessary for the assembly of the PVC complex. Scale bar, 100 nm. **b**, Modifications to *pvc13* or *pvc10* do not affect the loading of payload proteins. Endogenous PVC payloads Pdp1 and Pnf were tagged with HiBiT and loaded into either wild-type or mutant PVC complexes; successful payload loading was assessed with a HiBiT blot. No significant depletion of Pdp1 or Pnf was observed in the mutant PVCs, indicating these genes are not necessary for payload loading. **c**, PVC length regulation is not disrupted by modifications to *pvc13* or *pvc10*. PVC lengths were measured via a similar technique as in Extended Data Fig. 1b; no significant change in length distribution was observed in the mutant PVCs. **d**, PVCs bind to mouse macrophages in the absence of wild-type *pvc13* or *pvc10*. J774A.1 cells (a cell line used in previous studies of PVC activity^1–3^) were exposed to PVCs tagged with Flag (as in Fig. 1d) and binding was assessed with immunofluorescence. PVCs harbouring modified Pvc13 still clustered on the surface of J774A.1 cells, indicating this binding interaction was not mediated by Pvc13 in the same way that it was for human cells in Fig. 2a. Scale bar, 100 μm. **e**, PVC activity in mouse macrophages persists in the absence of wild-type *pvc13*. In support of the results from (d), PVCs containing truncated or deleted *pvc13* produced efficient cytotoxicity in J774A.1 cells, indicating PVC activity in this cell line is not the result of specific recognition by the tail fibre. However, deleting the spike tip (*pvc10*) does produce a loss of activity, suggesting the PVC may nonetheless be nonspecifically active against these cells. Values are mean ± s.d. with *n* = 3 biological replicates. Statistical significance was computed using one-way ANOVA with Bonferroni post hoc test; ****P < 0.0001; ns, not significant. Source data

**Extended Data Fig. 8:**
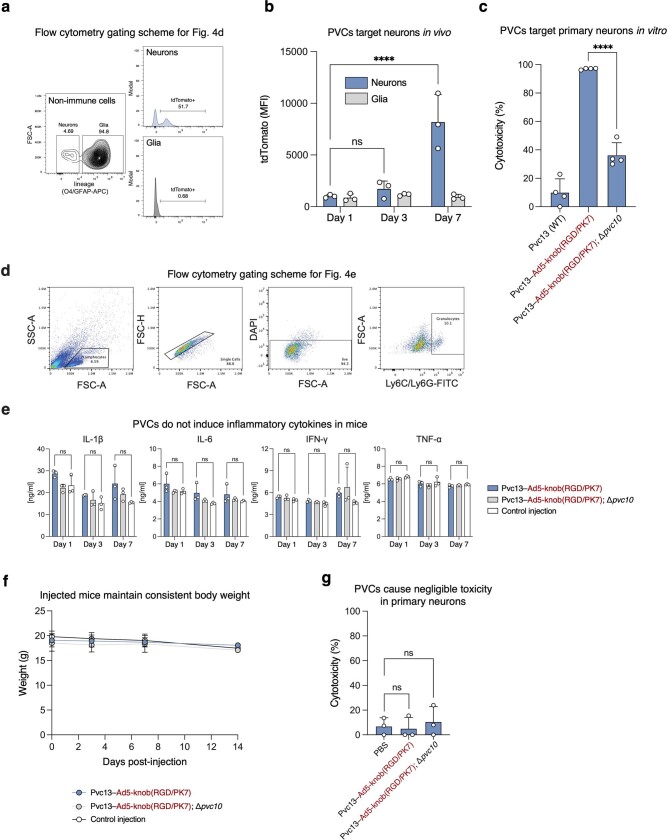
Additional evaluation of PVC tropism, immunogenicity and toxicity *in vivo*. **a**, Flow cytometry gating scheme for the *in vivo* tdTomato assay in Fig. 4d. **b**, Timecourse of PVC-induced tdTomato signal in the mouse brain after intracranial injection of PVCs (as in Fig. 4d). TdTomato signal in neurons began to increase at *t* = 3–7 days post-injection. **c**, PVCs can target primary neurons *in vitro*. Nearly complete cell death (97%) was observed, indicating PVCs equipped with Ad5 knob (RGD/PK7) exhibit robust tropism for neurons *in vitro*. **d**, Flow cytometry gating scheme for quantification of activated T cells and granulocytes in the immunogenicity assays from Fig. 4e. **e**, Quantification of inflammatory cytokines in the CNS after intracranial injection of PVCs with ELISA. No significant enrichment of any cytokine tested was observed (at *t* = 1, 3, or 7 days), indicating PVC injection does not stimulate a local inflammatory response in the CNS above background. **f**, Timecourse of animal weight after PVC injection. No significant loss of body weight was observed, indicating PVC injection likely does not produce significant systemic toxicity in these animals. **g**, Study of nonspecific cytotoxicity in primary neurons *in vitro*. PVCs loaded with Cre (in contrast to those in (c), which contained toxins) were added to cultured primary neurons and tested for cell death at *t* = 24 h. No significant cell death was observed above background, indicating these particles do not induce cellular toxicity in the absence of a toxin payload. All values (b,c, e–g) are mean ± s.d. with *n* = 3 (b, e–g) or *n* = 4 (c) biological replicates. Statistical significance was computed using either one-way ANOVA (c,g) or two-way ANOVA (b,e) with Bonferroni post hoc test; ****P < 0.0001; ns, not significant. Source data

After having identified novel PVC designs capable of targeting mouse cells, we next attempted to achieve protein delivery in vivo. We loaded Cre into PVCs harbouring Pvc13–Ad5-knob(RGD/PK7) and performed intracranial injections with the resulting particles in *loxP*-tdTomato reporter mice. We also injected separate mice with similar PVCs lacking a spike tip (Pvc10), a protein we found to be necessary for PVC-mediated delivery in vitro (Fig. 4b); we chose this design as a negative control for the in vivo experiments because Δ*pvc10* PVCs still form intact particles and load payloads (Extended Data Fig. 7a–c) and were found to produce less nonspecific activity in macrophages than Δ*pvc13* PVCs (Extended Data Fig. 7d,e). After intracranial injections with the Pvc13–Ad5-knob(RGD/PK7) particles, we observed Cre-mediated tdTomato expression in the hippocampus (Fig. 4c), indicating that the PVCs are active in vivo. Furthermore, we extracted single-cell suspensions from treated brains and quantified the tdTomato signal in neurons and microglia with flow cytometry (Fig. 4d and Extended Data Fig. 8a,b); we found a significant enrichment of tdTomato signal in neurons (but not microglia), indicating that PVCs harbouring Pvc13–Ad5-knob(RGD/PK7) can target neurons in vivo (we confirmed this result in vitro against primary neurons; Extended Data Fig. 8c). We also found that PVC treatment did not produce any significant activation of immune cells (Fig. 4e and Extended Data Fig. 8d), production of inflammatory cytokines (Extended Data Fig. 8e), loss of body weight (Extended Data Fig. 8f) or cellular toxicity (Extended Data Fig. 8g), indicating that PVC treatment was not immunogenic or toxic during this experimental time course. Finally, we also found that intact PVCs could be readily purified from treated brains at *t* = 0 or 1 day but not after *t* = 7 days (Fig. 4f), indicating that PVCs are transient in the brain and do not persist for extended periods of time; this suggests this system is ideally suited for therapies meant to be temporary or short-lived. Together, these results demonstrate that PVCs can deliver proteins in vivo, suggesting that this system is well-positioned for eventual use as a delivery tool for human use.

**Extended Data Fig. 9:**
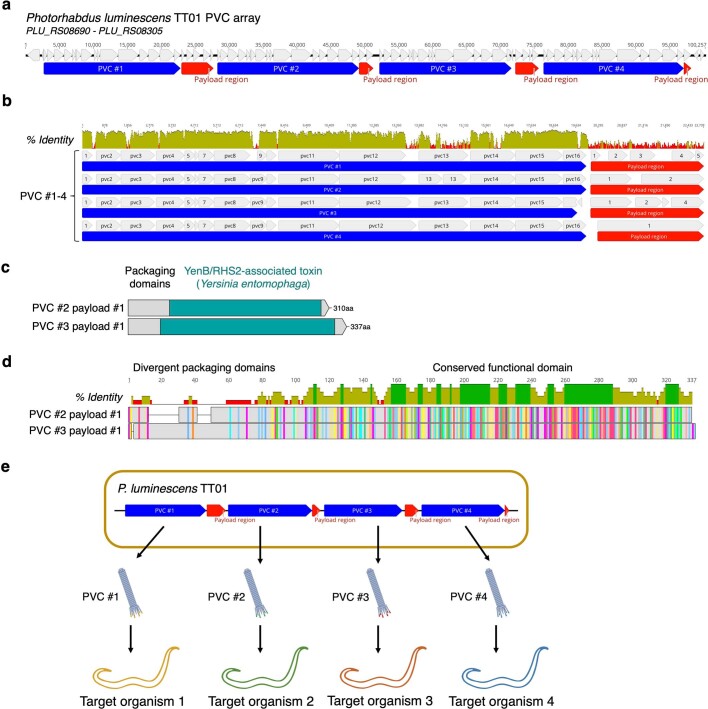
The PVC array in *P. luminescens* TT01 reveals a multiplexed protein delivery strategy. **a**, *P. luminescens* TT01 contains an array of four PVC loci in tandem on the genome, with each structural/accessory region (blue) immediately followed by putative payload genes (red). **b**, DNA sequence alignment of the four PVC loci in the *P. luminescens* TT01 PVC array. The loci share significant sequence homology but diverge at the target recognition gene (*pvc13*) and the payload region. This suggests these PVCs share a core structure but target different organisms and deliver different payloads to each target. Percent identity is presented with a sliding window of *n* = 50 bp. **c**, HHpred-predicted domain organization of two putative payload genes shared by separate PVC loci in the *P. luminescens* TT01 PVC array. While both genes share a common domain (YenB/RHS2-associated toxin; E = 0.0000067/0.0000039), both also contain unknown sequences on the N termini. Given that the PVCpnf payloads Pdp1 and Pnf are loaded via N-terminal packaging domains (Extended Data Fig. 3b–d), it is likely these N-terminal domains represent packaging domains which load these proteins into PVC complexes. **d**, Amino acid sequence alignment of putative payload proteins from (c). The two payloads share significant sequence homology in the functional domain (YenB/RHS2-associated toxin) but diverge at the N termini. It is plausible these two payloads are “sorted” into different PVC particles within *P. luminescens* TT01 via their divergent N-terminal packaging domains. Percent identity is presented with a sliding window of *n* = 5 aa. **e**, Proposed model for multiplexed protein delivery by *P. luminescens* TT01. This organism likely produces four distinct PVC particles, each with a different payload (sorted into each particle via unique packaging domains) and a different targeting apparatus (*pvc13*), which could be secreted to effect divergent biological activity within multiple host organisms (or cell types/tissues) simultaneously.

**Extended Data Fig. 10:**
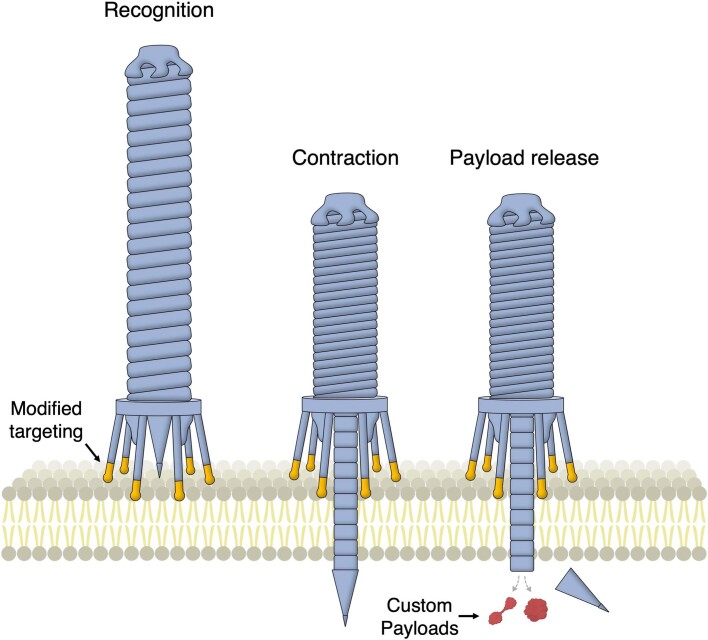
Model for protein delivery via reprogrammed PVCs. In this work, we demonstrated that PVCs can be engineered 1.) to load and deliver diverse novel payloads, enabling the introduction of novel biological activity into target cells, and 2.) to target novel organisms, enabling the delivery of human cells and live mice with high efficiency and specificity. We conclude that PVCs constitute a new class of highly programmable protein delivery devices.

In summary, we have demonstrated that an eCIS (PVCpnf) is a programmable protein delivery device that can be modified both to load non-native payloads (Fig. 1e,g) and to target novel organisms (Figs. 2a and 4b and Extended Data Figs. 5, 6, 9 and 10). Our studies of the PVC targeting element (*pvc13*; tail fibre) further showed that PVCs are highly target-specific and that PVC activity is dependent on the successful binding of the tail fibre with a receptor on the target cell (Figs. 2a,d and 3b–d and Extended Data Figs. 5d and 6e,f). Finally, we demonstrated the application of PVCs as delivery tools in diverse contexts, such as in the specific killing of cancer cells or as mediators of genome editing (Fig. 2a–d), and we showed that the system operates as intended in insect cells (Fig. 1f,g), human cells (Figs. 2 and 3), primary cells (Fig. 4b and Extended Data Fig. 8c) and in live mice (Fig. 4c,d and Extended Data Fig. 8b). Together, this work constitutes the development of a versatile class of programmable protein delivery tools that are well-suited for use in a variety of applications ranging from biocontrol to human gene therapy.

## Methods

### Plasmid construction

The PVCpnf structural and accessory region (*pvc1-16*) and payload and regulatory region (*Pdp1*, *Pnf* and regulatory genes *PAU_RS16570-RS24015*) were synthesized de novo (GenScript) and cloned into pAWP78 and pBR322 backbones, respectively. All manipulations involving payload and regulatory plasmids (pPayload) involved standard PCR amplification with Phusion Flash 2x Master Mix (ThermoFisher), assembly with either Gibson Assembly Master Mix (NEB E2611L) or Golden Gate Assembly with AarI and T4 DNA Ligase (ThermoFisher ER1582; NEB M0202), and transformation into chemically competent Stbl3 cells. PVC structural and accessory plasmids (pPVC) were amplified with KOD Xtreme Hot Start DNA Polymerase (Sigma-Aldrich 71975) with several modifications to the manufacturer’s protocol: 100 ng template DNA, 16 cycles and 30 min extension time. These plasmids were then assembled using Gibson Assembly Master Mix with 2–4 h incubation periods at 50 °C and electroporated into EPI300 electrocompetent cells (Lucigen EC300110). A summary of plasmids generated during this work can be found in Supplementary Table 7; annotated plasmid sequences can be found in Supplementary Data 1.

### PVC purification

For each PVC condition, one variant each of pPVC and pPayload were electroporated into EPI300 cells and PVC particles were purified using a modified version of a method used previously^9^. Colonies were inoculated into 2 ml Terrific Broth (US Biological T2810) and shaken at 37 °C for 16 h before being inoculated (at 1:1,000) into 500 ml TB medium and shaken at 30 °C for an additional 24 h. Cultures were then spun for 30 min at 4,000*g* and resuspended in 28 ml lysis buffer (25 mM Tris-HCl pH 7.5 (ThermoFisher 15567027), 140 mM NaCl (AmericanBio AB01915), 3 mM KCl (Sigma-Aldrich P9541), 5 mM MgCl_2_ (Sigma-Aldrich M4880), 200 μg ml^−1^ lysozyme (ThermoFisher 89833), 50 μg ml^−1^ DNase I (Sigma-Aldrich DN25), 0.5% Triton X-100 (Sigma-Aldrich 93443), and 1 × Protease Inhibitor Cocktail (MedChem Express HY-K0010)) and were subsequently shaken at 37 °C for 90 min to promote lysis. Lysates were then pelleted at 4,000*g* for 30 min at 4 °C to remove bulk cell lysate. Supernatants were then extracted and spun in an ultracentrifuge at 120,000*g* for 2 h at 4 °C to pellet PVC protein complexes. Pellets were resuspended in 1 ml PBS (Life Technologies 10010049) and spun at 16,000*g* for 15 min at 4 °C to remove residual solid lysate. Supernatants were then applied to 28 ml cold PBS before repeating the ultracentrifuge spin (120,000*g* for 2 h) and clarification spin (16,000*g* for 15 min) another 2 times. Final pellets were resuspended in 50 µl PBS and PVC yield was quantified by *A*_280_ measurement on a NanoDrop instrument (ThermoFisher). For mouse experiments, lipopolysaccharide was then removed from the final PVC samples using a detergent-based method^46^; in brief, samples were diluted into 1 ml cold PBS and 20 µl of Triton X-114 (Sigma-Aldrich X-114) was added. Samples were then incubated at 4 °C in a tube turner for 30 min, transferred to 37 °C for 10 min to allow the detergent to come out of solution, and spun at 20,000*g* for 20 min at 37 °C to separate the protein and detergent phases. The upper phase (containing the protein) was extracted and the procedure was repeated 2 more times (that is, Triton X-114 was added 3 times in total) and the final protein phase was incubated with 300 mg Bio-Beads SM-2 (Bio-Rad 1523920) at 4 °C in a tube turner overnight. Protein samples were then extracted from the beads, passed through a 0.2-μm sterile filter (Pall 4612), and concentrated down to 50 µl PBS with a final ultracentrifuge spin; endotoxin levels were then quantified using a Pierce Chromogenic Endotoxin Quant Kit (ThermoFisher A39552). All PVC samples were stored in PBS at 4 °C for a maximum of 1 week prior to use.

### Purification of PVC payloads

To determine whether endogenous PVC payloads (Pdp1 and Pnf) produced cytotoxicity independent of the PVC complex, we purified each of these proteins in isolation. Each payload was tagged with an affinity and solubility tag (6×His–Strep–SUMO) and was transformed into *E. coli* BL21 (DE3) competent cells (Sigma-Aldrich CMC0016). Colonies were inoculated into 5 ml TB medium and shaken at 37 °C for 16 h before being inoculated (at 1:200) into 1 l additional TB. These cultures were then shaken at 37 °C until they reached an *A*_600_ of 0.6–0.8, whereupon they were induced with 0.5 mM IPTG (Goldbio I2481C) and shaken at 37 °C for an additional 4 h. Cultures were then spun at 4,000*g* for 30 min and resuspended in 50 ml cold lysis buffer (50 mM Tris-HCl pH 7.5 (ThermoFisher 15567027), 280 mM NaCl (AmericanBio AB01915), 3 mM KCl (Sigma-Aldrich P9541), 5 mM MgCl_2_ (Sigma-Aldrich M4880), 1 µl benzonase (Sigma-Aldrich E1014) per 50 ml of buffer, and 1 tablet cOmplete (Sigma-Aldrich 11836170001) per 50 ml of buffer); resuspended cells were stirred for 30 min to ensure a homogenous mixture, and were then twice passed through a Microfluidics M110P microfluidizer. Lysates were then spun at 9,000*g* for 30 min at 4 °C and supernatants were applied to 2.5 ml of a 50% slurry (in lysis buffer) of Strep-Tactin Superflow Plus resin (Qiagen 30004) and stirred at 4 °C for 30 min. The resin was then pelleted at 2,000 *g* for 3 min at 4 °C, twice washed with 40 ml lysis buffer, and finally applied to a column (ThermoFisher 29922) and allowed to drain. With the column capped, we next added 12.5 ml of cold elution buffer (25 mM Tris-HCl pH 7.5 (ThermoFisher 15567027), 140 mM NaCl (AmericanBio AB01915), 3 mM KCl (Sigma-Aldrich P9541), 5 mM MgCl_2_ (Sigma-Aldrich M4880), and 100 µl per column of SUMO protease (a gift from J. Strecker)) and incubated the column overnight at 4 °C to liberate the protein from the resin. The purified protein was then concentrated using a 10 kDa Amicon Ultra filter (Sigma-Aldrich UFC901024), quantified by A280 measurement on a NanoDrop instrument (ThermoFisher), and verified for proper expression and purification by SDS–PAGE followed by Coomassie stain. Raw, uncropped versions of all protein gels can be found in Supplementary Fig. 1.

### Payload loading assays

To determine whether a protein was loaded into PVCs, we exploited the tendency of our PVC purification procedure to preferentially purify large molecular weight complexes over free proteins (Extended Data Fig. 3a). Payload proteins (cloned into pPayload) were tagged with C-terminal HiBiT tags and PVC particles containing the tagged payloads were purified. The baseplate of the PVC (encoded by *pvc12*) was also tagged with HiBiT to serve as a loading control for the western blot. Twenty micrograms of the resulting PVCs (containing loaded payloads) was then mixed with NuPAGE LDS Sample Buffer (ThermoFisher NP0008) and NuPAGE Sample Reducing Agent (ThermoFisher NP0009), both to a final concentration of 1×, and were subsequently boiled at 95 °C for 10 min. The denatured PVC payload samples were then run on NuPAGE Bis-Tris 1–12% protein gels (ThermoFisher NP0321) for 30 min at 200 V in 1× MOPS buffer (ThermoFisher NP000102) and were blotted onto PVDF membranes using an iBlot 2 instrument (ThermoFisher). To visualize low molecular weight payloads (as was done in Extended Data Fig. 3c,d), we instead ran the denatured protein samples on NuPAGE Bis-Tris 12% protein gels (ThermoFisher NP0342) in 1× MES buffer (ThermoFisher B0002). Finally, payload bands were visualized using the Nano-Glo HiBiT blotting system (Promega N2410) and images were captured with a Bio-Rad ChemiDoc instrument. A representative amino acid sequence of a non-native protein loaded via a PVC packaging domain can be found in Supplementary Table 5.

### Cell culture

A list of cell lines used in this study can be found in Supplementary Table 8. Cell lines were not authenticated or tested for *Mycoplasma* prior to use as they were primarily obtained from commercial sources. Unless otherwise stated, mammalian cells were maintained in T75 flasks (ThermoFisher 156499) at 37 °C with 5% CO_2_ in either DMEM-GlutaMAX (ThermoFisher 10569044) or RPMI-GlutaMAX (ThermoFisher 61870127), and insect cells were gently shaken in 125-ml shaker flasks (Sigma-Aldrich CLS431143) at 28 °C in ESF921 (VWR 100000-000). All media were supplemented with 10 µg ml^−1^ gentamicin (Sigma-Aldrich G1397) and 1× penicillin-streptomycin (ThermoFisher 15140122); mammalian media were also supplemented with 10% FBS (VWR 97068-085). For growth of primary splenocytes, the medium was also supplemented with mouse IL-2 (Peprotech 212-12) and 50 µM 2-mercaptoethanol (ThermoFisher 21985023).

### In vitro PVC delivery experiments

To detect PVC-mediated protein delivery in vitro, target cells were seeded into 96-well clear-bottom 96-well plates (VWR 89091-012) and allowed to grow to about 80% confluence. PVCs were then added to a final concentration of 150 ng µl^−1^ in 50 µl of cell culture medium per well. For assays involving co-transfection of a Cre reporter plasmid or a guide RNA plasmid, DNA was transfected immediately after adding PVCs using GeneJuice Transfection Reagent (Sigma-Aldrich 70967) for human cells or Insect GeneJuice Transfection Reagent (Sigma-Aldrich 71259) for Sf9 cells. For assays involving transfection of a target receptor (for example, EGFR or surface-displayed anti-epitope tag antibodies in Fig. 3), this was done 24 h prior to addition of PVCs. For toxin delivery experiments, cytotoxicity was assessed using CellTiter-Glo 2.0 Cell Viability Assay (Promega G9241) and/or staining with viability stain (8 ng µl^−1^ FDA (Sigma-Aldrich F7378) + 20 ng µl^−1^ PI (Sigma-Aldrich P4170)) and imaging under a Zeiss Observer D1 microscope; these analyses were carried out at *t* = 24 h for mammalian cells and *t* = 2 days (CellTiter-Glo)/4 days (FDA/PI stain and imaging) for Sf9 cells. For CellTiter-Glo assays, any wells exhibiting higher luminescence than the control well (PBS) were assigned a cytotoxicity value of 0% to avoid negative cytotoxicity. For assays involving Cre-driven GFP expression, cells were incubated for four days and were then imaged with a Leica DMi8 confocal microscope and analysed with flow cytometry (see ‘Flow cytometry analysis for in vitro PVC experiments’). For gene editing experiments, cells were incubated for 4 days, genomic DNA was extracted with 50 µl QuickExtract DNA Extraction Solution (Lucigen QE09050), and indels or base substitutions were quantified with NGS (see ‘Deep sequencing’). All numerical data from PVC experiments were plotted with Prism (9.3.1) and figures were graphically assembled in Adobe Illustrator (25.2.3).

### In silico protein structure prediction

To predict the structure of novel PVC tail fibre designs, we leveraged ColabFold, a Google Colab-based implementation of AlphaFold2^35–37^. For all tail fibre designs, sequences were queried as trimers in AlphaFold2_mmseqs2 (v1.2) with default model/MSA settings and num_recycles set to 12. Runs were supported by Google Cloud virtual machines running NVIDIA Tesla A100 GPUs. The resulting structures were visualized and recoloured with PyMOL (2.5.2).

### Electron microscopy

Routine negative-stain TEM analysis of purified PVC particles was performed either at the Koch Institute Nanotechnology Materials Core Facility or the MIT Materials Research Laboratory. In brief, 5–10 µl of each PVC sample (diluted to 100–500 ng µl^−1^) were applied to a glow discharged 200-mesh carbon-coated copper TEM grid (VWR 100489-722) for 60 s before removing excess liquid with a Kimwipe. Grids were then twice treated with 10 µl of 2% uranyl acetate stain (dabbing away the first immediately and the second after 30 s) or 5 times treated with 2% uranyl formate stain (incubating with gentle agitation for 5s, 5 s, 10 s, 30 s and 30 s) and allowed to dry at room temperature. Grids were then imaged in either a (1) JEOL 2100 FEG microscope at 200 kV equipped with a Gatan 2k × 2k UltraScan CCD camera, or a (2) FEI Tecnai (G2 Spirit TWIN) microscope at 120 kV equipped with a Gatan Orius SC1000B camera.

To determine whether PVC particles bind to target cells, we used a modified negative-stain TEM method. A549 cells were allowed to adhere at high density to glow discharged 200-mesh carbon-coated gold TEM grids (VWR 76499-704) in 24-well plates before being exposed to a high dose of PVC sample (1.8 µg µl^−1^ final concentration) for 3 h. The cells were then fixed for 10 min with 4% paraformaldehyde (Electron Microscopy Sciences 1574), washed once with PBS, 5× stained with 2% uranyl formate (via the same method as above), and allowed to dry at room temperature. The cells were then imaged with a FEI Tecnai (G2 Spirit TWIN) microscope at 120 kV equipped with a Gatan Orius SC1000B camera.

High-resolution imaging of PVC-treated human cells was conducted using scanning electron microscopy (SEM). A549 cells were grown to 80–90% confluence on 12-mm glass coverslips (VWR 354087) in 24-well plates before being exposed to a moderate dose of PVC sample (500 ng µl^−1^) for 3 h. The cells were then fixed for 1 h with 2.5% glutaraldehyde/2% paraformaldehyde/100 mM sodium cacodylate at 4 °C, rinsed twice with 100 mM sodium cacodylate (each for 5 min at 4 °C), treated with 1% osmium tetroxide/sodium cacodylate for 30 min at 4 °C, rinsed 3–4 times (10 min each) with distilled water, dehydrated with ethanol, treated with 50% TMS/50% ethanol for 15 min, treated with 80% TMS/20% ethanol for 15 min, twice treated with 100% TMS for 5 min each, and allowed to air dry before sputter coating and imaging in a Zeiss Crossbeam 540 SEM/focused ion beam.

### Immunofluorescence

To determine whether PVC particles bound to target cells, we tagged an external PVC protein (Pvc2) with an N-terminal Flag tag and exposed the resulting PVC particles (at 300 ng µl^−1^) to target cells for 3 h at 37 °C. The cells were then fixed for 10 min with 4% paraformaldehyde (Electron Microscopy Sciences 1574), blocked for 1 h with blocking buffer (10% goat serum (Sigma-Aldrich G9023) and 0.1% Triton X-100 (Sigma-Aldrich 93443) diluted in PBS), stained for 1 h with M2 anti-Flag antibody (Sigma-Aldrich F1804; diluted 1:500 in blocking buffer), stained for 1 hr with an Alexa Fluor 488-conjugated secondary antibody (ThermoFisher A11001; diluted 1:1,000 in blocking buffer), stained for 10 min with 1 µg ml^−1^ DAPI (ThermoFisher D1306; diluted in PBS), and imaged using a Leica DMi8 confocal microscope. An amino acid sequence depicting the position of the Flag tag on Pvc2 can be found in Supplementary Table 6.

We also used immunofluorescence to examine the effect of PVCs on the cytoskeleton (Extended Data Fig. 6a). Target cells were first seeded into 96-well plates and allowed to grow to about 80% confluence before being exposed to PVCs (150 ng µl^−1^ final concentration) for 24 h. The cells were then fixed for 10 min with 4% paraformaldehyde, blocked for 1 h with blocking buffer, stained for 1 h with rhodamine phalloidin (ThermoFisher R415; diluted to 1× final concentration in blocking buffer), stained for 10 min with 1 µg ml^−1^ DAPI (ThermoFisher D1306; diluted in PBS), and imaged using a Leica DMi8 confocal microscope.

### Flow cytometry analysis for in vitro PVC experiments

For experiments involving PVC-mediated delivery of Cre, we measured delivery efficiency using flow cytometry. Cells were first harvested by incubation with TrypLE Express dissociation reagent (ThermoFisher 12604), pelleted at 300*g* for 3 min, and resuspended in 100 µl of flow cytometry buffer (PBS supplemented with 2% EDTA (Life Technologies 15575020) and 5% FBS (VWR 97068-085)). Samples were run on a Beckman Coulter Cytoflex S flow cytometer, and analysis was performed using CytExpert (2.3.1.22) and FlowJo (10.8.2). A representative scheme for gating and threshold setting is shown in Extended Data Fig. 4c.

### Deep sequencing

To detect PVC-induced genomic edits in target cells, we first amplified the target region out of each genomic DNA extract (see ‘In vitro PVC delivery experiments’) with NEBNext High-Fidelity 2× PCR Master Mix (NEB M0541). Target regions were then barcoded with indexed Illumina P5 and P7 NGS primers. Libraries were purified with a Qiagen PCR Purification Kit (Qiagen 28104), quantified on a NanoDrop instrument (ThermoFisher), and sequenced on an Illumina MiSeq instrument (with read length set to 300 bp). Indels and base substitutions were then quantified with Geneious Prime (2020.0.5). Primers used for deep sequencing can be found in Supplementary Table 9.

### Quantitative PCR

To assess the effect of regulatory genes on PVC gene expression, we used quantitative reverse transcription PCR (RT-qPCR). *E. coli* EPI300 cells were electroporated with one variant each of pPVC and pPayload (as described in ‘PVC purification’) and colonies were shaken in 5 ml TB at 37 °C for 16 h. The cultures were then spun for 5 min at 4,000*g*, resuspended in 750 µl TRI reagent (Zymo R2073), incubated at room temperature for 5 min, and mechanically lysed by vortexing (1 min) with 250 µl of 0.5 mm Zirconia beads (Fisher NC0450473). We then added 200 µl chloroform, incubated at room temperature for 3 min, spun for 15 min at 12,000*g* (4 °C), and extracted the aqueous phase for RNA extraction via a Zymo Direct-zol RNA Miniprep Kit (Zymo R2073) with the optional DNAse step. We then generated cDNA from these bulk RNA extracts using ProtoScript II Reverse Transcriptase (NEB M0368) and random primers (NEB S1330) with the manufacturer’s protocol. Finally, we ran qPCR on the resulting cDNAs using Fast SYBR Green Master Mix (ThermoFisher 4385612) in a Bio-Rad CFX Opus 384 qPCR instrument. Delta-delta Ct values were computed against the housekeeping gene *gapA*^47^. Primers used for qPCR can be found in Supplementary Table 10.

### Mass spectrometry

PVCs were diluted to about 36 μg µl^−1^ in PBS before being sent to the Koch Institute Biopolymers and Proteomics Facility for analysis by mass spectrometry. In brief, proteins were reduced with 10 mM dithiothreitol (Sigma-Aldrich 11583786001) for 10 min at 95 °C and then alkylated with 20 mM iodoacetamide (Sigma-Aldrich I5161) for 30 min at 25 °C in the dark. Proteins were then digested with trypsin on S-Trap micro columns (ProtiFi C02-micro-80) per the manufacturer’s protocol. The tryptic peptides were separated by reverse-phase HPLC (Thermo UltiMate 3000) using a PepMap RSLC C18 column and a 2 μm EASY-Spray tip (ThermoFisher ES903) over a 90-min gradient before being subjected to nano-electrospray using an Exploris mass spectrometer (Thermo). The resulting mapped peptide hits can be found in Supplementary Data 2.

### Intracranial injections

All mouse experiments conformed to guidelines established by the National Institutes of Health and were conducted under protocols approved by the Institutional Animal Care and Use Committee (IACUC) of the Broad Institute of MIT and Harvard. Animals were chosen randomly for treatment with either control or experimental conditions without blinding. Female Ai9 mice (aged 8–12 weeks) were obtained from the Jackson Laboratory (strain 007909). All mice were maintained on a 12-h light:dark cycle with ad libitum access to food and water. Mice were anaesthetized using isoflurane (2–3%) and prepared for stereotaxic surgery; fur was shaved, and mice were placed in a stereotaxic frame (Kopf Instruments). A heating pad was placed under the mice to prevent hypothermia. Isoflurane (1–2%) was delivered via a nose cone throughout the surgery. Ophthalmic ointment was used to protect the eyes. Buprenorphine -SR (1 mg kg^−1^, subcutaneous) was given before the start of surgery. Bupivacaine (1 mg kg^−1^) was injected intradermally along the incision line as a form of local anaesthetic. Meloxicam (2 mg kg^−1^) was also administered subcutaneously prior to surgery. The scalp was disinfected with betadine scrub and 70% ethanol. An incision was made using a scalpel along the scalp midline. The exposed skull was thoroughly cleaned, and a craniotomy was made above the hippocampus. PVC proteins were targeted to the hippocampus (−2.3 AP, 1.25 ML, −3 & −1.5 DV), and slowly pressure-injected (100 nl min^−1^) using a 10 µl Hamilton syringe (700 Series Microliter Syringes, Hamilton, Model 701 N Syringe) and a micro-syringe pump controller (Micro 4; WPI). After injection, the needle was left in place for 2 min and then slowly withdrawn. A total of 1,000 nl (Fig. 4c; 500 nl at −2.0 DV and 500 nl at −1.5 DV) at 7.5 µg µl^−1^ or 3,000 nl (Fig. 4d–f and Extended Data Fig. 8b,e,f; 1,500 nl at −3.0 DV and 1,500 nl at −1.5 DV) at 1.2 µg µl^−1^ of PVC sample was injected per mouse. After injection, the skin was sealed with a simple, continuous suture pattern with 4-0 Ethilon nylon sutures. Incisions were swabbed clean with 0.9% sterile saline and sterile cotton tip applicators. Mice were postoperatively hydrated with saline and housed in a temperature-controlled environment until achieving an ambulatory recovery. To relieve post-operative pain, meloxicam (2 mg kg^−1^) was administered subcutaneously every 24 h up to a minimum of 72 h post-surgery.

### Imaging of mouse brain sections

At *t* = 12 days post-injection, mice were deeply anaesthetized with Fatal-Plus at a dose of 90 mg kg^−1^ and transcardially perfused with 20 ml of PBS, followed by 20 ml of 4% paraformaldehyde solution. Brains were quickly extracted and stored in 4% paraformaldehyde solution at 4 °C for 24 h, and were then transferred to 30% sucrose in PBS solution and allowed to equilibrate for 2 days. Brains were then mounted on a cryostat using OCT and sectioned coronally (50 µm). The floating sections were washed in PBS and stained for neurons using anti-NeuN antibody (Sigma-Aldrich MAB377; 1:500) and Alexa 488 secondary antibody (ThermoFisher A11001; 1:1,000). The sections were mounted on slides with PVA-DABCO. Images were acquired using a Leica DMi8 confocal microscope with a 10× and 20× air objective.

### Isolation and flow cytometry of PVC-injected neurons

Animals were deeply anaesthetized after *t* = 1, 3 and 7 days with CO_2_ and transcardially perfused with 20 ml of PBS. Brains of PVC- or mock-injected mice were extracted, and targeted hemispheres were cut into pieces using scalpels and digested with 50 µg ml^−1^ liberase (Sigma-Aldrich 05401119001) at 37 °C for 30 min. Single-cell suspensions were generated using slow repetitive pipetting. Myelin was manually removed using Myelin Removal Beads II, human, mouse, rat (Miltenyi Biotec 130-096-733) and LS columns (Miltenyi Biotec 130-042-401) followed by enrichment of neuronal cells using the adult neuron isolation kit (Miltenyi Biotec 130-126-603) and LS columns. Enriched cell populations were fixed using Cytofix Fixation Buffer (BD 554655) at 4 °C for 30 min and blocked with 1:50 TruStain FcX (anti-mouse CD16/32) reagent (BioLegend 101320) prior to antibody staining for flow cytometry; antibodies and dilutions can be found in Supplementary Table 12.

### Isolation and culture of mouse primary neurons for in vitro PVC targeting

Ninety-six-well plates were coated with 0.05 mg ml^−1^ poly-d-lysine (BD 354210) one day prior to isolation. A dissection solution was made using HBSS (ThermoFisher 14025092) supplemented with 10 mM HEPES (ThermoFisher 15630080), 33 mM d-glucose (Sigma-Aldrich G8270) and 43 mM sucrose (Sigma-Aldrich S0389). Timed-pregnant female C57BL/6J mice (aged 12 weeks) were killed according to the standard operating procedures of the Institutional Animal Care and Use Committees (IACUC) of the Broad Institute of MIT and Harvard. Brains were extracted from embryos at embryonic day 16.5 and dissected in dissection solution. Pan-cortex tissue was used for downstream neuron isolation. Tissues were digested using TrypLE Select (ThermoFisher 12563011) for 30 min and washed twice in dissection solution supplemented with trypsin inhibitor (Sigma-Aldrich T9253) and BSA (Sigma-Aldrich A9418). Single-cell suspension was prepared by repetitive trituration and cells were cultured in Neurobasal-A Medium (ThermoFisher 10888022) supplemented with B-27 Plus Supplement (ThermoFisher A3582801).

### Assessment of in vivo CNS inflammation

Isolation of CNS-infiltrating myeloid and T cells was performed as previously described^48^. In brief, mice were deeply anaesthetized after *t* = 1, 3 and 7 days with CO_2_ and transcardially perfused with 20 ml of PBS. Brains of PVC- or mock-injected mice were extracted, and targeted hemispheres were cut into pieces using scalpels and digested with 50 µg ml^−1^ liberase (Sigma-Aldrich 05401119001) at 37 °C for 30 min and subsequently mashed through 100-µm and 70-µm cell strainers (Greiner One-Bio 542000 and 542070). Myelin was removed using a 30% continuous Percoll (Sigma-Aldrich GE17-0891-01) gradient and density centrifugation at 2,700 rpm. Following myelin removal, single-cell suspension of brain-infiltrating immune cells were prepared in PBS and blocked with 1:50 TruStain FcX (anti-mouse CD16/32) reagent (BioLegend 101320) prior to antibody staining for flow cytometry. DAPI staining solution (Miltenyi Biotec 130-111-570) was added at 1:100 dilution immediately prior to flow cytometry analysis to discriminate live cells. Interstitial fluid surrounding the parenchymal cells of the brain was isolated by washout of minced brain tissue at indicated post-injection timepoints in PBS and centrifugation at 500*g*. Cytokine ELISAs for interleukin-1β (IL-1β), interleukin-6 (IL-6), interferon-γ (IFN-γ) and tumour necrosis factor (TNF) were performed according to the manufacturer’s protocol (Invitrogen 88-7013-22, 88-7064-22, 88-7314-22 and 88-7324-22, respectively) and absorbance at 450–570 nm was measured. Cytokine concentrations were calculated corresponding to diluted standards as per the manufacturer’s protocol.

### In vivo PVC clearance assay

To study the persistence of PVCs in the mouse brain, interstitial fluid was isolated from brain homogenates. In brief, PVC-treated mice were euthanized and transcardiac perfusion with PBS was performed prior to extraction of full intact brains. Brain tissue was mechanically dissociated using sterile scalpels followed by dounce homogenization into single-cell suspensions. These single-cell suspensions were centrifuged at 500 *g* for 5 min, and the clarified supernatants were diluted into 28 ml PBS and ultracentrifuged at 120,000*g* for 2 h at 4 °C to pellet any intact PVC protein complexes. Pellets were resuspended in 50 µl PBS and spun at 16,000*g* for 15 min at 4 °C to remove residual bulk homogenate. Finally, we analysed the resuspensions with negative-stain TEM to detect intact PVC complexes; see ‘Electron microscopy’.

### Statistics and reproducibility

All statistical analyses were performed in Prism (9.3.1). Quantitative data are presented as mean ± s.d. with *n* = 2–4 biological replicates per condition; the number of replicates presented are listed in the figure legends. Unless otherwise stated, biological replicates represent independent treatments in separate culture wells or mice. All micrographs, gels, and blots are representative images from at least *n* = 3 independent experiments. Statistical significance was computed using one-way or two-way ANOVA followed by Bonferroni post hoc tests (to correct for multiple comparisons), as indicated in the figure legends. *P* values below 0.05 were considered statistically significant; the results of all statistical tests (including *P* values) are included in the Source Data alongside the associated source data for each figure panel.

### Reporting summary

Further information on research design is available in the Nature Portfolio Reporting Summary linked to this article.

## Online content

Any methods, additional references, Nature Portfolio reporting summaries, source data, extended data, supplementary information, acknowledgements, peer review information; details of author contributions and competing interests; and statements of data and code availability are available at 10.1038/s41586-023-05870-7.

### Supplementary information

Supplementary Information Supplementary Fig. 1 and Supplementary Tables 1–12.

Reporting Summary

Supplementary Data 1 Annotated sequence files for plasmids listed in Supplementary Table 7.

Supplementary Data 2 Mass spectrometry analysis of a purified PVC sample.

### Source data

Source Data Fig. 1

Source Data Fig. 2

Source Data Fig. 3

Source Data Fig. 4

Source Data Extended Data Fig. 1

Source Data Extended Data Fig. 2

Source Data Extended Data Fig. 4

Source Data Extended Data Fig. 6

Source Data Extended Data Fig. 7

Source Data Extended Data Fig. 8

## Data Availability

All plasmids generated during this work (Supplementary Table 7) are available from Addgene. Sequencing reads are available from the Sequence Read Archive under BioProject ID PRJNA929529. Uncropped gel and immunoblot images can be found in Supplementary Fig. 1. Source data are provided with this paper. All additional data are available from the authors upon request.
