# Supplementary material for: Programmable protein delivery with a bacterial contractile injection system
Source: Nature. 2023 Mar 29;616(7956):357–64. doi: 10.1038/s41586-023-05870-7 (PMC10097599; doi:10.1038/s41586-023-05870-7)
Supplement: Supplementary file 1 — Supplementary Fig. 1 and Supplementary Tables 1–12. [file 41586_2023_5870_MOESM1_ESM.pdf]

nature portfolio

https://doi.org/10.1038/s41586-023-05870-7

Supplementary information

Programmable protein delivery with a bacterial contractile injection system

In the format provided by the authors and unedited

Supplementary Information

Programmable protein delivery with a bacterial contractile injection system

Joseph Kreitz[1,2,3,4,5], Mirco J. Friedrich[1,2,3,4,5], Akash Guru[1,2,3,4,5], Blake Lash[1,2,3,4,5], Makoto Saito[1,2,3,4,5], Rhiannon K Macrae[1,2,3,4,5], and Feng Zhang[1,2,3,4,5]†

(1) Howard Hughes Medical Institute, Cambridge, MA 02139, USA
(2) Broad Institute of MIT and Harvard, Cambridge, MA 02142, USA
(3) McGovern Institute for Brain Research at MIT, Cambridge, MA 02139, USA
(4) Department of Brain and Cognitive Science, Massachusetts Institute of Technology, Cambridge, MA 02139, USA
(5) Department of Biological Engineering, Massachusetts Institute of Technology, Cambridge, MA 02139, USA

† Correspondence should be addressed to F.Z. (zhang@broadinstitute.org).

Table of contents

Supplementary Figures……………………………………………………………………3
Supplementary Tables……………………………………………………………………5
Supplementary Data………………………………………………………………….....13
References…………………………………………………………………………………..14

Supplementary Figures

Supplementary Figure 1 | Uncropped images for all gels and immunoblots depicted in the main figures and Extended Data figures.

a, Coomassie gel from Fig. 1c depicting purified PVC complexes.

b, Immunoblot from Fig. 1e depicting Pdp1 and non-native payloads (GFP, Cre, and a ZFN) loaded into PVC particles. Because Pvc12 (baseplate) is found in equal multiplicity between each PVC complex, we also stained against this protein to serve as a loading control for this blot.

c, Coomassie gel from Extended Data Fig. 1e verifying the importance of regulatory genes (*PAU_RS16570-RS24015*) in the recovery of PVC complexes from *E. coli*.

d, Immunoblot from Extended Data Fig. 1h showing that a structural component of the PVC (Pvc12) is still expressed in the absence of *PAU_RS16570-RS24015*.

e, Immunoblots from Extended Data Fig. 4b used for dose determination in the experiment of Extended Data Fig. 4a

f, Immunoblot from Extended Data Fig. 3c depicting the loading properties of truncated variants of Pdp1. As in (b), Pvc12 was used as a loading control for this blot.

g, Immunoblot from Extended Data Fig. 3d depicting the loading properties of truncated variants of Pnf. Note that because Pnf is more sparse in the sample than Pvc12 or Pdp1, to visualize the Pnf bands while avoiding signal saturation in the Pvc12 bands we used two different exposure times for this figure; this is noted in the figure margins. As in (b) and (f), Pvc12 was also used as a loading control for this blot.

h, Immunoblots from Extended Data Fig. 5b implicating the CTD of Pvc13 as the target recognition domain. Because our purification method removes unloaded proteins (see Extended Data Fig. 3a), the presence of payload protein in all three conditions (shown via a separate Cre blot) confirmed these samples all contained assembled PVC complexes.

i, Immunoblot from Extended Data Fig. 7b confirming the three negative control PVC designs used throughout the study (Δ*pvc13*, truncated *pvc13*, and Δ*pvc10*) still load payload proteins.

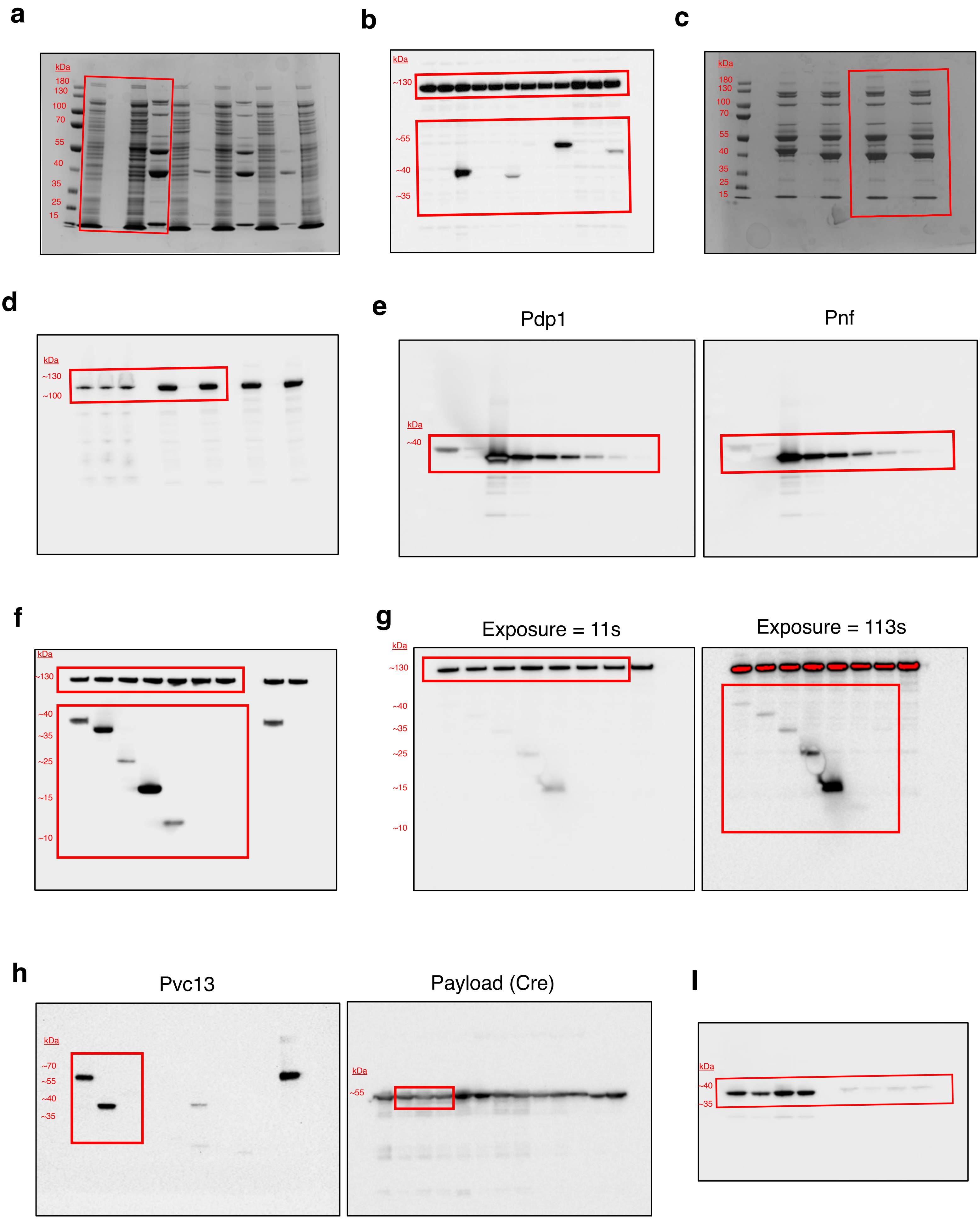
a
kDa
180
130
100
70
55
40
35
25
15
b
kDa
~130
~55
~40
~35
c
kDa
180
130
100
70
55
40
35
25
15
d
kDa
~130
~100
e
Pdp1
Pnf
kDa
~40
f
kDa
~130
~40
~35
~25
~15
~10
g
Exposure = 11s
Exposure = 113s
kDa
~130
~40
~35
~25
~15
~10
h
Pvc13
Payload (Cre)
kDa
~70
~55
~40
~35
kDa
~55
I
kDa
~40
~35

Supplementary Tables

Supplementary Table 1 | Amino acid sequence of a PVC tail fiber retargeted with an expanded host-range Ad5 knob domain.

- Pvc13(NTD+shaft) - 2xGGSGG linker - Ad5 knob domain (with RGD and PK7 modifications) - 2xGGSGG linker - Pvc13(CTD)

MNETRYNATVQEQQTLSNPKAVGPDIDKLKDKFKEGSIPLQTDFNELIDIADIGRKACGQAPQQ
NGPGEGLKLADDGTLNLKIGTFSNKDFSPLILKDDVLSVDLGSGLTNETNGICVGQGDGITVNT
SNVAVKQGNGISVTSSGGVAVKVSANKGLSVDSSGVAVKVNTDKGISVDGNGVAVKVNTSKGIS
VDNTGVAVIANASKGISVDGSGVAVIANTSKGISVDGSGVAVIANTSKGISVDNTGVAVIANAS
KGISVDGSGVAVIANTSKGISVDGSGVAVIANTSKGISVDSSGVAVKVKANGGIKVDANGVAID
PNNVLPKGVIVMFSGSTAPTGWALCDGNNGTPNLIDRFILGGKGTDINGVSTNTASGTKNSKLF
DFSSDEATLTIDGKTLGRGGSGGGGSGGNDKLTLWTTPAPSPNCRLNAEKDAKLTLVLTKCGSQ
ILATVSVLAVKGSLAPISGTVQSAHLIIRFDENGVLLNNSFLDPEYWNFRNGDLTEGTAYTNAV
GFMPNLSAYPKSHGKTAKSNIVSQVYLNGDKTKPVTLTITLNGTQETGDTTACDCRGDCFCGPS
AYSMSFSWDWSGHNYINEIFATSSYTFSYIAQEGSGSGSGSGSKKKKKKGGSGGGGSGGHDHD
IKITGTGKHSHKNKVTVPYYILAFIIKL

Supplementary Table 2 | Amino acid sequence of a PVC tail fiber retargeted with an anti-EGFR DARPin.

- Pvc13(NTD+shaft) - 2xGGSGG linker - E01 anti-EGFR DARPin - 2xGGSGG linker - Pvc13(CTD)

MNETRYNATVQEQQTLSNPKAVGPDIDKLKDKFKEGSIPLQTDFNELIDIADIGRKACGQAPQQ
NGPGEGLKLADDGTLNLKIGTFSNKDFSPLILKDDVLSVDLGSGLTNETNGICVGQGDGITVNT
SNVAVKQGNGISVTSSGGVAVKVSANKGLSVDSSGVAVKVNTDKGISVDGNGVAVKVNTSKGIS
VDNTGVAVIANASKGISVDGSGVAVIANTSKGISVDGSGVAVIANTSKGISVDNTGVAVIANAS
KGISVDGSGVAVIANTSKGISVDGSGVAVIANTSKGISVDSSGVAVKVKANGGIKVDANGVAID
PNNVLPKGVIVMFSGSTAPTGWALCDGNNGTPNLIDRFILGGKGTDINGVSTNTASGTKNSKLF
DFSSDEATLTIDGKTLGRGGSGGGGSGGDLGKKLLEAARAGQDDEVRILMANGADVNADDTWGW
TPLHLAAYQGHLEIVEVLLKNGADVNAYDYIGWTPLHLAADGHLEIVEVLLKNGADVNASDYIG
DTPLHLAAHNGHLEIVEVLLKHGADVNAQDKFGKTAFDISIDNGNEDLAEILQGGSGGGGSGGH
DHDIKITGTGKHSHKNKVTVPYYILAFIIKL

Supplementary Table 3 | Amino acid sequence of a PVC tail fiber retargeted with an anti-MHCII nanobody (Nb).

- Pvc13(NTD+shaft) - 6xGGSGG linker - VHH7 anti-MHCII Nb - 2xGGSGG linker - Pvc13(CTD)

MNETRYNATVQEQQTLSNPKAVGPDIDKLKDKFKEGSIPLQTDFNELIDIADIGRKACGQAPQQ
NGPGEGLKLADDGTLNLKIGTFSNKDFSPLILKDDVLSVDLGSGLTNETNGICVGQGDGITVNT
SNVAVKQGNGISVTSSGGVAVKVSANKGLSVDSSGVAVKVNTDKGISVDGNGVAVKVNTSKGIS
VDNTGVAVIANASKGISVDGSGVAVIANTSKGISVDGSGVAVIANTSKGISVDNTGVAVIANAS
KGISVDGSGVAVIANTSKGISVDGSGVAVIANTSKGISVDSSGVAVKVKANGGIKVDANGVAID
PNNVLPKGVIVMFSGSTAPTGWALCDGNNGTPNLIDRFILGGKGTDINGVSTNTASGTKNSKLF
DFSSDEATLTIDGKTLGRGGSGGGGSGGGGSGGGGSGGGGSGGGGSGGQVQLQESGGGLVQAGD
SLRLSCAASGRTFSRGVMGWFRRAPGKEREFVAIFSGSSWSGRSTYYSDSVKGRFTISRDNAKN
TVYLQMNGLKPEDTAVYYCAAGYPEAYSAYGRESTYDYWGQGTQVTVSSGGSGGGGSGGHDHDI
KITGTGKHSHKNKVTVPYYILAFIIKL

Supplementary Table 4 | Amino acid sequence of a PVC tail fiber retargeted with an HA tag.

- Pvc13(NTD+shaft) - 1xGGSGG linker - HA tag - 1xGGSGG linker - Pvc13(CTD)

MNETRYNATVQEQQTLSNPKAVGPDIDKLKDKFKEGSIPLQTDFNELIDIADIGRKACGQAPQQ
NGPGEGLKLADDGTLNLKIGTFSNKDFSPLILKDDVLSVDLGSGLTNETNGICVGQGDGITVNT
SNVAVKQGNGISVTSSGGVAVKVSANKGLSVDSSGVAVKVNTDKGISVDGNGVAVKVNTSKGIS
VDNTGVAVIANASKGISVDGSGVAVIANTSKGISVDGSGVAVIANTSKGISVDNTGVAVIANAS
KGISVDGSGVAVIANTSKGISVDGSGVAVIANTSKGISVDSSGVAVKVKANGGIKVDANGVAID
PNNVLPKGVIVMFSGSTAPTGWALCDGNNGTPNLIDRFILGGKGTDINGVSTNTASGTKNSKLF
DFSSDEATLTIDGKTLGRGGSGGYPYDVPDYAGGSGGHDHDIKITGTGKHSHKNKVTVPYYILA
FIIKL

Supplementary Table 5 | Amino acid sequence of a protein payload (zinc finger deaminase) modified for loading and delivery via a PVC.

- Pdp1_NTD - 3xGGSGG linker - FLAG - SV40 NLS - ZFD

MPRYANYQINPKQNIKNSHGKSSSSDFSSGYLSFSNNSLDDPFIRQQVKREFIWEGHMKEIEEA
SRLGGSGGGGSGGGGSGGDYKDDDDKDMAPKKKRKVGIHGVPAAMGGSYALGPYQISAPQLPAY
NGQTVGTFYYVNDAGGLESKVFSSGGPTPYPNYANAGHVEGQSALFMRDNGISEGLVFHNNPEG
TCGFCVNMTETLLPENAKMTVVPPEGSGTPHEVGVYTLSGTPHEVGVYTLFQCRICMRKFATSG
SLTRHTKIHTGEKPFQCRICMRNFSRSDHLSTHIRTHTGEKPFACDICGRKFATSSNRTKHTKI
HTHPRAPIPKPFQCRICMRNFSRSDNLSEHIRTHTGEKPFACDICGRKFAWHSSLRVHTKIHLR
SGGSTNLSDIIEKETGKQLVIQESILMLPEEVEEVIGNKPESDILVHTAYDESTDENVMLLTSD
APEYKPWALVIQDSNGENKIKML

Supplementary Table 6 | Amino acid sequence of the PVC sheath protein (Pvc2) tagged with FLAG for IF detection of PVC particles.

- FLAG - G - Pvc2

MDYKDDDDKGTTVTSYPGVYIEELNSLALSVSNSATAVPVFAVDEQNQYISEDNAIRINSWMDY
LNLIGNFNNEDKLDVSVRAYFANGGGYCYLVKTTSLEKIIPTLDDVTLLVAAGEDIKTTVDVLC
QPGKGLFAVFDGPETELTINGAEEAKQAYTATPFAAVYYPWLKADWANIDIPPSAVMAGVYASV
DLSRGVWKAPANVALKGGLEPKFLVTDELQGEYNTGRAINMIRNFSNTGTTVWGARTLEDKDNW
RYVPVRRLFNSVERDIKRAMSFAMFEPNNQPTWERVRAAISNYLYSLWQQGGLAGSKEEDAYFV
QIGKGITMTQEQIDAGQMIVKVGLAAVRPAEFIILQFTQDVEQR

Supplementary Table 7 | Summary of plasmids used in this study (annotated GenBank files provided in Supplementary Data 1).

ID	Plasmid name	Type	Source	Description
1	pAWP78-PVCpnf1-16	pPVC	This work	Unmodified pPVC; contains WT PVC structural/accessory genes
2	pBR322-PVCpnf17-22	pPayload	This work	Unmodified pPayload; contains WT PVC payloads/regulatory genes
3	pBR322-PVCpnf18-21	pPayload	This work	Contains only regulatory genes; for generating empty PVCs
4	pBR322-Pdp1_NTD-Cre	pPayload	This work	For loading Cre into PVCs; also contains regulatory genes
5	pBR322-Pdp1_NTD-Cas9	pPayload	This work	For loading SpCas9 into PVCs; also contains regulatory genes
6	pBR322-Pdp1_NTD-ZFD_L	pPayload	This work	For loading L half of zinc finger deaminase targeting human TRAC locus (ZFD_TRAC-NC-Left-G1397-N_DddA)[4]; also contains regulatory genes
7	pBR322-Pdp1_NTD-ZFD_R	pPayload	This work	For loading R half of zinc finger deaminase targeting human TRAC locus (ZFD_TRAC-NC-Right-G1397-C_DddA)[4]; also contains regulatory genes
8	pIEx4-Dio-GFP	N/A	This work	Cre reporter for insect cells; double-floxxed-EGFP; generated using RV-Cag-Dio-GFP[5]
9	pcDNA3-Dio-GFP	N/A	This work	Cre reporter for mammalian cells; double-floxxed-EGFP; generated using RV-Cag-Dio-GFP[5]
10	pIEx4-Cre	N/A	This work	Expresses Cre in insect cells; positive control for Cre-GFP assay; for threshold setting in flow cytometry
11	pcDNA3-Cre	N/A	This work	Expresses Cre in mammalian cells; positive control for Cre-GFP assay; for threshold setting in flow cytometry
12	py100-sgRNA_hVEGFA	N/A	This work	Expression of SpCas9 guide RNA specific for human VEGFA locus
13	pAWP78-PVCpnfΔpvc13	pPVC	This work	Produces PVCs lacking tail fibers
14	pAWP78-PVCpnfΔpvc15	pPVC	This work	Produces PVCs lacking payloads by elimination of the payload loader (*pvc15*)

15	pAWP78-PVCpnf_FLAG-pvc2	pPVC	This work	Contains an external epitope tag on the sheath protein; for immunofluorescence-based binding assays
16	pAWP78-PVCpnf_pvc13-truncated	pPVC	This work	Contains a tail fiber with a truncated binding domain (lacking aa403-476 in Pvc13); produces nontargeting PVCs
17	pAWP78-PVCpnf_pvc13-E01DARPin	pPVC	This work	Produces PVCs retargeted with anti-EGFR DARPin E01[6]
18	pAWP78-PVCpnf_pvc13-A4DARPin	pPVC	This work	Produces PVCs retargeted with anti-lysozyme DARPin A4[7]; used as a negative control for DARPin retargeting method
19	pAWP78-PVCpnf_pvc13-Ad5Knob	pPVC	This work	Produces PVCs retargeted with knob domain from hAd5 virus
20	pAWP78-PVCpnf_pvc13-Ad5DL491-492	pPVC	This work	Produces PVCs retargeted with non-binding mutant of Ad5 knob[8]; used as a negative control for Ad5 knob retargeting method
21	pAWP78-PVCpnf_pvc13-Ad5RGDPK7	pPVC	This work	Produces PVCs retargeted with expanded tropism hAd5 knob (RGD and PK7 insertions)[9]
22	pAWP78-PVCpnf_pvc13-antiMouseMHCIINb	pPVC	This work	Produces PVCs retargeted with VHH7 anti-mouse MHC class II nanobody[10]
23	pAWP78-PVCpnf_pvc13-antiHistoneNb	pPVC	This work	Produces PVCs retargeted with A9 anti-histone nanobody[11]; used as a negative control for Nb retargeting method
24	pAWP78-PVCpnf_pvc13-CD4DARPin	pPVC	This work	Produces PVCs retargeted with anti-CD4 DARPin D27.2[12]; for killing Jurkat cells
25	pAWP78-PVCpnf_pvc13-CD11bDARPin	pPVC	This work	Produces PVCs retargeted with anti-CD11b DARPin F7[13]; negative control for Jurkat killing assay
26	pAWP78-PVCpnf_pvc13-HA	pPVC	This work	Produces PVCs retargeted with HA tag
27	pAWP78-PVCpnf_pvc13-FLAG	pPVC	This work	Produces PVCs retargeted with FLAG tag
28	pAWP78-PVCpnf_pvc13-EE	pPVC	This work	Produces PVCs retargeted with EE tag
29	pAWP78-PVCpnf_pvc13-MoonTag	pPVC	This work	Produces PVCs retargeted with MoonTag[14]
30	pAWP78-PVCpnf_pvc13-SunTag	pPVC	This work	Produces PVCs retargeted with SunTag[15]
31	pAWP78-PVCpnf_pvc13-ALFA	pPVC	This work	Produces PVCs retargeted with ALFA tag[16]

32	pHCMV-SS-antiHAscFv-TM	N/A	Gift from D. Strebinger	Displays anti-HA scFv[17] from the surface of mammalian cells
33	pHCMV-SS-antiFLAGscFv-TM	N/A	This work	Displays anti-FLAG scFv[18] from the surface of mammalian cells
34	pHCMV-SS-antiEEscFv-TM	N/A	This work	Displays anti-EE scFv[19] from the surface of mammalian cells
35	pHCMV-SS-antiMoonTagNb-TM	N/A	This work	Displays anti-MoonTag nanobody[14] from the surface of mammalian cells
36	pHCMV-SS-antiSunTag-TM	N/A	This work	Displays anti-SunTag scFv[15] from the surface of mammalian cells
37	pHCMV-SS-antiALFAtagNb-TM	N/A	This work	Displays anti-ALFA nanobody[16] from the surface of mammalian cells
38	pAWP78-PVCpnf_pvc12-HiBiT	pPVC	This work	Produces PVCs tagged with HiBiT on the baseplate; used as assembly/loading control for payload loading assays
39	pAWP78-PVCpnfΔpvc15_pvc12-HiBiT	pPVC	This work	Negative control for payload loading assay; produces PVCs deficient for payload loader (*pvc15*)
40	pBR322-Pdp1-HiBiT	pPayload	This work	Loads HiBiT-tagged Pdp1 into PVCs; for payload loading assays; also contains Pnf
41	pBR322-Pdp1_aa1-289-HiBiT	pPayload	This work	Loads a truncated payload; for identification of Pdp1 packaging domain
42	pBR322-Pdp1_aa1-190-HiBiT	pPayload	This work	Loads a truncated payload; for identification of Pdp1 packaging domain
43	pBR322-Pdp1_aa1-133-HiBiT	pPayload	This work	Loads a truncated payload; for identification of Pdp1 packaging domain
44	pBR322-Pdp1_aa1-76-HiBiT	pPayload	This work	Loads a truncated payload; for identification of Pdp1 packaging domain
45	pBR322-Pdp1_aa1-38-HiBiT	pPayload	This work	Loads a truncated payload; for identification of Pdp1 packaging domain
46	pBR322-Pdp1_aa39-331-HiBiT	pPayload	This work	Loads a truncated payload; for identification of Pdp1 packaging domain
47	pBR322-Pdp1_NTD-GFP-HiBiT	pPayload	This work	Loads HiBiT-tagged GFP into PVCs using Pdp1 packaging domain; also contains Pnf
48	pBR322-Pdp1_NTD-Cre-HiBiT	pPayload	This work	Loads HiBiT-tagged Cre into PVCs using Pdp1 packaging domain; also contains Pnf
49	pBR322-Pdp1_NTD-ZFN_L-HiBiT	pPayload	This work	Loads HiBiT-tagged L fragment of a zinc finger nuclease specific for human AAVS1 locus[20] using Pdp1 packaging domain

50	EGFR-GFP	N/A	Ref. 21	Displays EGFR from the surface of mammalian cells; used to sensitize EGFR-deficient cells to EGFR-targeting PVCs
51	TwinStrep-SUMO-Pdp1	N/A	This work	For affinity purification of Pdp1 payload
52	TwinStrep-SUMO-Pnf	N/A	This work	For affinity purification of Pnf payload
53	pAWP78-PVCpnfΔpvc10	pPVC	This work	Produces PVCs containing a truncated spike tip protein (Pvc10)
54	pAWP78-PVCpnfΔpvc10_ pvc13-Ad5RGDPK7	pPVC	This work	Produces PVCs retargeted with expanded tropism hAd5 knob (RGD and PK7 insertions)[9], also containing a truncated spike tip protein (Pvc10)
55	pAWP78-PVCpnfΔpvc10_ pvc13-antiMouseMHCIINb	pPVC	This work	Produces PVCs retargeted with VHH7 anti-mouse MHC class II nanobody[10], also containing a truncated spike tip protein (Pvc10)

Supplementary Table 8 | Cell lines used in this study.

Cell line	Organism	Source	Media
Sf9	Insect (*S. frugiperda*)	Sigma-Aldrich 71104M	ESF921
A549	Human (*H. sapiens*)	ATCC CCL-185	RPMI+GlutaMAX
U2OS	Human (*H. sapiens*)	ATCC HTB-96	DMEM+GlutaMAX
HEK293FT	Human (*H. sapiens*)	ThermoFisher R70007	DMEM+GlutaMAX
A549-LoxP-GFP	Human (*H. sapiens*)	Gift from D. Strebinger	RPMI+GlutaMAX
Jurkat	Human (*H. sapiens*)	ATCC TIB-152	RPMI+GlutaMAX
N2a	Mouse (*M. musculus*)	ATCC CCL-131	DMEM+GlutaMAX
NIH/3T3	Mouse (*M. musculus*)	ATCC CRL-1658	DMEM+GlutaMAX
A20	Mouse (*M. musculus*)	ATCC TIB-208	RPMI+GlutaMAX
J774A.1	Mouse (*M. musculus*)	ATCC TIB-67	DMEM+GlutaMAX
Primary splenocytes	Mouse (*M. musculus*)	Gift from B. Lash	RPMI+GlutaMAX

Supplementary Table 9 | NGS primer sequences.

Target	Guide sequence	NGS primer sequences
Hs Vegfa	ggtgagtgagtgtgtgcgtg	F: tcttcgagagtgaggacgtgtg
		R: tattggaatcctggagtgaccc
Hs Trac	N/A	F: ctgggacatgcaagcccataac
		R: tctcagagcttaggatgcacgc

Supplementary Table 10 | RT-qPCR primer sequences.

Target	F primer sequence	R primer sequence
pvc1	tggttcaaaatgccgggtcaga	aacagttctgttttccccgg
pvc2	gggggattagctggcagcaaag	gccctgcatcaatctgctcctg
pvc3	caaaatggcggaggagcttgct	cagatcgagttccgggcaaacc
pvc4	gtccgtatcgacagtttggccg	atgcctgttgcccgccattatc
pvc5	tttaaccgcattcccgatccgc	acgggcattttctcctccctca
pvc6	ggtagtgccatcaacacgaccg	cccaagtcgcctcatccagact
pvc7	accccccattgaagagcagctca	ttcccaacgcattttgccccat
pvc8	acttttggccgcggtagatgtg	cctacctgacagtgggccagtt
pvc9	gtcgtggttgggcttttcctcc	ataagacgttcccccggctcag
pvc10	aggcccttcagtgtaccagtgg	gtaacatccggttgtggcggag
pvc11	caccagagtgccagcattggag	gccatctcttgcagacgagcag
pvc12	ggctcagtgaatgatgacgccc	cgctgtgtgccaaagtaaccca
pvc13	agagggcagtattcccctgcaa	cgcttgaccacaggctttacgt
pvc14	agcccgttgcattacctgaaca	cggcattaaactgggcctgacg
pvc15	gctgaccacattggcgttgact	gcatcctgttccgccaaatcga
pvc16	ggtaatcgcccacggctttctc	gaagaaaccgcggtaaccggag
Pdp1	aaaaactggccgccttttgggt	ggctgagagactcgcttccaga
PAU_RS16570	tggaggaaggttagtttcac	acaggcccatagttccaggcaa
PAU_RS16565	gggggaagggtaaatgagaata	cgagtttttcgggggcaattat
PAU_RS16560	acagtcaccgatttgccgaagc	ccttcttctgttaaagccaatccc
PAU_RS24015	ggctgttttcattcacctcagtcaca	atttcgagttgcagcacgacgg
Pnf	gccctttcgccggatagtgaac	tcagcactcccggttttcccat
gapA[22]	gattacatggcatacatgctg	cagacgaacggtcaggtcaa

Supplementary Table 11 | Endotoxin measurements for PVCs used in the immune response assays in Fig. 4e and Extended Data Fig. 8e-f.

Sample	Endotoxin concentration (EU/mL)
PVC (Ad5 RGD/PK7)	0.674
PVC (Ad5 RGD/PK7) Δ*pvc10*	0.422
Control injection	0.500

Supplementary Table 12 | Antibodies used for flow cytometry during mouse experiments.

Epitope	Conjugate	Order number	Manufacturer	Dilution used
Mouse CD45	BV510	103138	BioLegend	1:200
Mouse/human CD11b	APC	101212	BioLegend	1:50
Mouse Ly-6G/Ly-6C (Gr-1)	FITC	108406	BioLegend	1:100
Mouse O4	APC	130-119-982	Miltenyi Biotec	1:100
Mouse GFAP	Alexa Fluor 647	51-9792-82	ThermoFisher Scientific	1:100
Mouse CD3	BV650	100229	BioLegend	1:200
Mouse CD4	PerCP	553052	BD	1:100
Mouse CD8	APC-Cy7	560182	BD	1:100

Supplementary Data

Supplementary Data 1 (separate file) | Annotated sequence files for plasmids listed in Supplementary Table 7.

Supplementary Data 2 (separate file) | Mass spectrometry analysis of a purified PVC sample.

References

1. Peng, K., Radivojac, P., Vucetic, S., Dunker, A. K. & Obradovic, Z. Length-dependent prediction of protein intrinsic disorder. *BMC Bioinformatics* **7**, 208 (2006).
2. Will, E. *et al.* Unmodified Cre recombinase crosses the membrane. *Nucleic Acids Res.* **30**, e59 (2002).
3. Kirby, I. *et al.* Identification of Contact Residues and Definition of the CAR-Binding Site of Adenovirus Type 5 Fiber Protein. *J. Virol.* **74**, 2804–2813 (2000).
4. Lim, K., Cho, S.-I. & Kim, J.-S. Nuclear and mitochondrial DNA editing in human cells with zinc finger deaminases. *Nat. Commun.* **13**, 366 (2022).
5. Ciceri, G. *et al.* Lineage-specific laminar organization of cortical GABAergic interneurons. *Nat. Neurosci.* **16**, 1199–1210 (2013).
6. Steiner, D., Forrer, P. & Plückthun, A. Efficient Selection of DARPins with Sub-nanomolar Affinities using SRP Phage Display. *J. Mol. Biol.* **382**, 1211–1227 (2008).
7. Fischer, G., Hogan, B.J., Houlihan, G., Edmond, S., Huovinen, T.T.K., Hollfelder, F., Hyvonen, M. Designed Ankyrin Repeat Protein (DARPin) A4 in complex with Lysozyme. (2018).
8. Kirby, I. *et al.* Mutations in the DG Loop of Adenovirus Type 5 Fiber Knob Protein Abolish High-Affinity Binding to Its Cellular Receptor CAR. *J. Virol.* **73**, 9508–9514 (1999).
9. Wu, H. *et al.* Double Modification of Adenovirus Fiber with RGD and Polylysine Motifs Improves Coxsackievirus–Adenovirus Receptor-Independent Gene Transfer Efficiency. *Hum. Gene Ther.* **13**, 1647–1653 (2002).
10. Rashidian, M. *et al.* Noninvasive imaging of immune responses. *Proc. Natl. Acad. Sci.* **112**, 6146–6151 (2015).

11. Moeglin, E. *et al.* A Novel Nanobody Precisely Visualizes Phosphorylated Histone H2AX in Living Cancer Cells under Drug-Induced Replication Stress. *Cancers* **13**, 3317 (2021).

12. Schweizer, A. *et al.* CD4-Specific Designed Ankyrin Repeat Proteins Are Novel Potent HIV Entry Inhibitors with Unique Characteristics. *PLoS Pathog.* **4**, e1000109 (2008).

13. Siegel, P. M. *et al.* A DARPin targeting activated Mac-1 is a novel diagnostic tool and potential anti-inflammatory agent in myocarditis, sepsis and myocardial infarction. *Basic Res. Cardiol.* **116**, 17 (2021).

14. Boersma, S. *et al.* Multi-Color Single-Molecule Imaging Uncovers Extensive Heterogeneity in mRNA Decoding. *Cell* **178**, 458-472.e19 (2019).

15. Tanenbaum, M. E., Gilbert, L. A., Qi, L. S., Weissman, J. S. & Vale, R. D. A protein tagging system for signal amplification in gene expression and fluorescence imaging. *Cell* **159**, 635–646 (2014).

16. Götzke, H. *et al.* The ALFA-tag is a highly versatile tool for nanobody-based bioscience applications. *Nat. Commun.* **10**, 4403 (2019).

17. Zhao, N. *et al.* A genetically encoded probe for imaging nascent and mature HA-tagged proteins in vivo. *Nat. Commun.* **10**, 2947 (2019).

18. Liu, Y. *et al.* Visualizing looping of two endogenous genomic loci using synthetic zinc-finger proteins with anti-FLAG and anti-HA frankenbodies in living cells. *Genes Cells Devoted Mol. Cell. Mech.* **26**, 905–926 (2021).

19. Kalyoncu, S. *et al.* Effects of protein engineering and rational mutagenesis on crystal lattice of single chain antibody fragments. *Proteins* **82**, 1884–1895 (2014).

20. Hockemeyer, D. *et al.* Highly efficient gene targeting of expressed and silent genes in human ESCs and iPSCs using zinc finger nucleases. *Nat. Biotechnol.* **27**, 851–857 (2009).

21. Carter, R. E. & Sorkin, A. Endocytosis of functional epidermal growth factor receptor-green fluorescent protein chimera. *J. Biol. Chem.* **273**, 35000–35007 (1998).

22. Jandu, N. *et al.* Enterohemorrhagic Escherichia coli O157:H7 Gene Expression Profiling in Response to Growth in the Presence of Host Epithelia. *PLOS ONE* **4**, e4889 (2009).
