# Supplementary material for: Programmable protein delivery with a bacterial contractile injection system
Source: Nature. 2023 Mar 29;616(7956):357–64. doi: 10.1038/s41586-023-05870-7 (PMC10097599; doi:10.1038/s41586-023-05870-7)
Supplement: Supplementary file 2 — Reporting Summary [file 41586_2023_5870_MOESM2_ESM.pdf]

nature portfolio

Corresponding author(s): Feng Zhang

Last updated by author(s): Feb 5, 2023

Reporting Summary

Nature Portfolio wishes to improve the reproducibility of the work that we publish. This form provides structure for consistency and transparency in reporting. For further information on Nature Portfolio policies, see our Editorial Policies and the Editorial Policy Checklist.

Statistics

For all statistical analyses, confirm that the following items are present in the figure legend, table legend, main text, or Methods section.

n/a	Confirmed	
☐	☒	The exact sample size (*n*) for each experimental group/condition, given as a discrete number and unit of measurement
☐	☒	A statement on whether measurements were taken from distinct samples or whether the same sample was measured repeatedly
☐	☒	The statistical test(s) used AND whether they are one- or two-sided<br>*Only common tests should be described solely by name; describe more complex techniques in the Methods section.*
☒	☐	A description of all covariates tested
☐	☒	A description of any assumptions or corrections, such as tests of normality and adjustment for multiple comparisons
☐	☒	A full description of the statistical parameters including central tendency (e.g. means) or other basic estimates (e.g. regression coefficient) AND variation (e.g. standard deviation) or associated estimates of uncertainty (e.g. confidence intervals)
☐	☒	For null hypothesis testing, the test statistic (e.g. *F*, *t*, *r*) with confidence intervals, effect sizes, degrees of freedom and *P* value noted<br>*Give P values as exact values whenever suitable.*
☒	☐	For Bayesian analysis, information on the choice of priors and Markov chain Monte Carlo settings
☒	☐	For hierarchical and complex designs, identification of the appropriate level for tests and full reporting of outcomes
☒	☐	Estimates of effect sizes (e.g. Cohen's *d*, Pearson's *r*), indicating how they were calculated

Our web collection on statistics for biologists contains articles on many of the points above.

Software and code

Policy information about availability of computer code

Data collection	We used AlphaFold2 (ColabFold mmseqs2 v1.2) to predict 3-dimensional protein structures.
Data analysis	We used Geneious Prime (2020.0.5) to quantify indels/base substitutions and to generate alignments, PyMOL (2.5.2) to visualize protein structures, Prism (9.3.1) to generate data plots and run statistical tests, CytExpert (2.3.1.22) and FlowJo (10.8.2) to analyze flow cytometry data, ImageJ (2.1.0) to analyze PVC lengths and gel band intensities, and Adobe Illustrator (25.2.3) to generate figures.

For manuscripts utilizing custom algorithms or software that are central to the research but not yet described in published literature, software must be made available to editors and reviewers. We strongly encourage code deposition in a community repository (e.g. GitHub). See the Nature Portfolio guidelines for submitting code & software for further information.

Data

Policy information about availability of data

All manuscripts must include a data availability statement. This statement should provide the following information, where applicable:

- Accession codes, unique identifiers, or web links for publicly available datasets
- A description of any restrictions on data availability
- For clinical datasets or third party data, please ensure that the statement adheres to our policy

All plasmids listed in Supplementary Table 7 are available from Addgene. Sequencing reads are available from the SRA database under BioProject ID PRJNA929529.

Uncropped gel and immunoblot images can be found in Supplementary Figure 1. The source data underlying all main figure and Extended Data figures, as well as the results of statistical analyses (including P values), are provided in the Source Data file. All additional data are available from the authors upon request.

Human research participants

Policy information about studies involving human research participants and Sex and Gender in Research.

Reporting on sex and gender	N/A - we did not use human research participants.
Population characteristics	N/A - we did not use human research participants.
Recruitment	N/A - we did not use human research participants.
Ethics oversight	N/A - we did not use human research participants.

Note that full information on the approval of the study protocol must also be provided in the manuscript.

Field-specific reporting

Please select the one below that is the best fit for your research. If you are not sure, read the appropriate sections before making your selection.

☒ Life sciences ☐ Behavioural & social sciences ☐ Ecological, evolutionary & environmental sciences

For a reference copy of the document with all sections, see nature.com/documents/nr-reporting-summary-flat.pdf

Life sciences study design

All studies must disclose on these points even when the disclosure is negative.

Sample size	Sample size (n) was set to 3 where possible. Several experiments involved sample sizes of 2 to simplify the technical procedure; these experiments are noted in the figure margins. No a priori calculations of sample size were performed; sample sizes were chosen by best practice and literature precedent. For example, Jiang et al. 2022 (Ref. 3) used n=3 for their PVC translocation assays (similar to our Fig. 1f-g); Lim, Cho, & Kim 2022 (Ref. 42) used either n=2 or n=3 for experiments involving ZFDs (which we used in Fig. 2c); and Rocchi et al. 2019 (Ref. 1) used n=3 for their cytotoxicity assays (similar to our Fig. 1f).
Data exclusions	No data were excluded from this study.
Replication	All replicates represent biological replicates (independent treatments in separate wells or animals) with n = 2-4 (as described above). All micrographs, gels, and blots are representative images from at least n = 3 independent experiments.
Randomization	For in vivo experiments, animals were chosen randomly for treatment with either control or experimental conditions. Sample randomization for all other experiments was not applicable as they involved cultured cells; all conditions received equivalent numbers of cells in separate wells.
Blinding	Blinding was not performed.

Reporting for specific materials, systems and methods

We require information from authors about some types of materials, experimental systems and methods used in many studies. Here, indicate whether each material, system or method listed is relevant to your study. If you are not sure if a list item applies to your research, read the appropriate section before selecting a response.

Materials & experimental systems

n/a	Involved in the study	
☐	☒	Antibodies
☐	☒	Eukaryotic cell lines
☒	☐	Palaeontology and archaeology
☐	☒	Animals and other organisms
☒	☐	Clinical data
☒	☐	Dual use research of concern

Methods

n/a	Involved in the study	
☒	☐	ChIP-seq
☐	☒	Flow cytometry
☒	☐	MRI-based neuroimaging

March 2021

Antibodies

Antibodies used	Anti-FLAG M2 antibody (Sigma-Aldrich F1804, Lot SLCG2330; 1:500), Anti-NeuN Antibody, clone A60 (Sigma-Aldrich MAB377, Lot 3856137; 1:500), Goat anti-Mouse IgG (H+L) Alexa Fluor 488 (ThermoFisher A11001, Lot 2247988; 1:1000), Mouse CD45 (BioLegend 103138, lot B360620; 1:200), Mouse/human CD11b (BioLegend 101212, lot B368966; 1:50), Mouse Ly-6G/Ly-6C (Gr-1) (BioLegend 108406, lot B363794; 1:100), Mouse O4 (Miltenyi Biotec 130-119-982, lot 1322110764; 1:100), Mouse GFAP (ThermoFisher 51-9792-82, lot 2497614; 1:100), Mouse CD3 (BioLegend 100229, lot B350667; 1:200), Mouse CD4 (BD 553052, lot B283419; 1:100), Mouse CD8 (BD 560182, lot 2168072; 1:100).
Validation	All antibodies were purchased from commercial sources and have been validated by the manufacturers. Verification statements and literature citations can be found at the manufacturers' websites:<br>Anti-FLAG M2 antibody: https://www.sigmaaldrich.com/US/en/product/sigma/f1804<br>Anti-NeuN antibody: https://www.emdmillipore.com/US/en/product/Anti-NeuN-Antibody-clone-A60,MM_NF-MAB377<br>Goat anti-Mouse IgG (H+L) Alexa Fluor 488: https://www.thermofisher.com/antibody/product/Goat-anti-Mouse-IgG-H-L-Cross-Adsorbed-Secondary-Antibody-Polyclonal/A-11001<br>Mouse CD45: https://www.biolegend.com/en-us/products/brilliant-violet-510-anti-mouse-cd45-antibody-7995?GroupID=BLG1932<br>Mouse/human CD11b: https://www.biolegend.com/en-us/products/apc-anti-mouse-human-cd11b-antibody-345<br>Mouse Ly-6G/Ly-6C (Gr-1): https://www.biolegend.com/en-us/products/fitc-anti-mouse-ly-6g-ly-6c-gr-1-antibody-458<br>Mouse O4: https://www.miltenyibiotec.com/US-en/products/o4-antibody-anti-human-mouse-rat-reafinity-rea576.html#pe:100-tests-in-200-ul<br>Mouse GFAP: https://www.thermofisher.com/antibody/product/GFAP-Antibody-clone-2-2B10-Monoclonal/51-9792-82<br>Mouse CD3: https://www.biolegend.com/en-us/search-results/brilliant-violet-650-anti-mouse-cd3-antibody-7843<br>Mouse CD4: https://www.bdbiosciences.com/en-us/products/reagents/flow-cytometry-reagents/research-reagents/single-color-antibodies-ruo/percp-rat-anti-mouse-cd4.553052<br>Mouse CD8: https://www.bdbiosciences.com/en-us/products/reagents/flow-cytometry-reagents/research-reagents/single-color-antibodies-ruo/apc-h7-rat-anti-mouse-cd8a.560182

Eukaryotic cell lines

Policy information about cell lines and Sex and Gender in Research

Cell line source(s)	All cell lines are listed in Supplementary Table 8.<br>Cell lines from ATCC: A549, U2OS, Jurkat, N2a, NIH/3T3, A20, J774A.1<br>Cell lines from ThermoFisher: HEK293FT<br>Cell lines from Sigma-Aldrich: Sf9<br>Cell lines from colleagues: A549-LoxP-GFP, primary splenocytes (from female Ai9 mice)
Authentication	None of these cell lines were authenticated prior to use.
Mycoplasma contamination	None of these cell lines were tested for Mycoplasma prior to use.
Commonly misidentified lines<br>(See ICLAC register)	No commonly misidentified cell lines were used in this study.

Animals and other research organisms

Policy information about studies involving animals; ARRIVE guidelines recommended for reporting animal research, and Sex and Gender in Research

Laboratory animals	Specific pathogen-free facilities at the Broad Institute was used for the storage and care of all mice. Mice were housed at a temperature of 67–73°F, relative humidity of 30–60%, and maintained in a 12h light–dark cycle. Timed-pregnant Female C57BL/6J mice (strain 000664) aged 12 weeks were used for embryonal neuron isolation and female Ai9 (B6.Cg-Gt(ROSA)26Sortm9(CAG-tdTomato)Hze/J) mice (strain 007909) aged 8–12 weeks were purchased from the Jackson Laboratory and used for intracranial brain injection experiments and tissue isolation.
Wild animals	No wild animals were used in this study.
Reporting on sex	Embryonal neuron isolation is only possible from pregnant female mice. As we did not expect a difference in PVC tropism between males and females (since all endogenous receptors targeted in this study are present in both male and female tissues), brain injections were restricted to female animals.
Field-collected samples	We did not collect samples from the field.
Ethics oversight	All mouse experiments conformed to guidelines established by the National Institutes of Health and were conducted under protocols approved by the Institutional Animal Care and Use Committees (IACUC) of the Broad Institute of MIT and Harvard.

Note that full information on the approval of the study protocol must also be provided in the manuscript.

Flow Cytometry

Plots

Confirm that:

☒ The axis labels state the marker and fluorochrome used (e.g. CD4-FITC).

☒ The axis scales are clearly visible. Include numbers along axes only for bottom left plot of group (a 'group' is an analysis of identical markers).

☒ All plots are contour plots with outliers or pseudocolor plots.

☒ A numerical value for number of cells or percentage (with statistics) is provided.

Methodology

Sample preparation	Cells were first harvested by incubation with TrypLE Express dissociation reagent (ThermoFisher 12604), pelleted at 300 g for 3min, and resuspended in 100 µL of flow cytometry buffer [PBS supplemented with 2% EDTA (Life Technologies 15575020) and 5% FBS (VWR 97068-085)]. Additional details pertaining to flow cytometry methods can be found in the sections entitled "Flow cytometry analysis for in vitro PVC experiments" and "Isolation and flow cytometry of PVC-infected neurons".
Instrument	Samples were run on a Beckman Coulter Cytoflex S flow cytometer.
Software	Analysis was performed using CytExpert and FlowJo software.
Cell population abundance	Post-sort fractions were not analyzed.
Gating strategy	Representative schemes for gating and threshold setting is shown in Extended Data Fig. 4c, 8a, and 8d. For the in vitro experiments, we first removed debris from the raw data by plotting FFC/SSC and selecting the cluster that exhibited FITC signal in a positive control condition (transfected Cre and LoxP-GFP). We set a GFP-/+ threshold roughly halfway between the GFP- peak and nascent GFP+ peak. We then applied this threshold to experimental data to determine the proportion of cells expressing GFP.

☒ Tick this box to confirm that a figure exemplifying the gating strategy is provided in the Supplementary Information.
